# Comparative performance of the BGISEQ-500 vs Illumina HiSeq2500 sequencing platforms for palaeogenomic sequencing

**DOI:** 10.1093/gigascience/gix049

**Published:** 2017-06-26

**Authors:** Sarah Siu Tze Mak, Shyam Gopalakrishnan, Christian Carøe, Chunyu Geng, Shanlin Liu, Mikkel-Holger S Sinding, Lukas F K Kuderna, Wenwei Zhang, Shujin Fu, Filipe G Vieira, Mietje Germonpré, Hervé Bocherens, Sergey Fedorov, Bent Petersen, Thomas Sicheritz-Pontén, Tomas Marques-Bonet, Guojie Zhang, Hui Jiang, M Thomas P Gilbert

**Affiliations:** 1 Centre for GeoGenetics, Natural History Museum of Denmark, University of Copenhagen, Øster Voldgade 5-7, 1350 Copenhagen, Denmark; 2 DTU Bioinformatics, Department of Bio and Health Informatics, Technical University of Denmark, Building 208, DK-2800 Lyngby, Denmark; 3 BGI-Shenzhen, Shenzhen 518083, China; 4 China National GeneBank, BGI-Shenzhen, Shenzhen 518083, China; 5 Natural History Museum, University of Oslo, PO Box 1172 Blindern, N-0318 Oslo, Norway; 6 The Qimmeq Project, University of Greenland, Manutooq 1, PO Box 1061, 3905 Nuussuaq, Greenland; 7 Institute of Evolutionary Biology (UPF-CSIC), PRBB, Dr. Aiguader 88, 08003 Barcelona, Spain; 8 CNAG-CRG, Centre for Genomic Regulation (CRG), Barcelona Institute of Science and Technology (BIST), Baldiri i Reixac 4, 08028 Barcelona, Spain; 9 OD Earth and History of Life, Royal Belgian Institute of Natural Sciences, Vautierstraat 29, 1000 Brussels, Belgium; 10 Department of Geosciences, Palaeobiology, University of Tübingen, Tübingen, Germany; 11 Senckenberg Centre for Human Evolution and Palaeoenvironment, University of Tübingen, Tübingen, Germany; 12 Mammoth Museum, Institute of Applied Ecology of the North of the North-Eastern Federal University, ul. Kulakovskogo 48, 677980 Yakutsk, Russia; 13 Catalan Institution of Research and Advanced Studies (ICREA), Passeig de Lluís Companys, 23, 08010, Barcelona, Spain; 14 Centre for Social Evolution, Department of Biology, Universitetsparken 15, University of Copenhagen, Copenhagen DK-2100, Denmark; 15 Trace and Environmental DNA Laboratory, Department of Environment and Agriculture, Curtin University, 6102 Perth, Australia; 16 Norwegian University of Science and Technology, University Museum, 7491 Trondheim, Norway

**Keywords:** ancient DNA, BGISEQ-500, Illumina HiSeq 2500, comparative performance

## Abstract

Ancient DNA research has been revolutionized following development of next-generation sequencing platforms. Although a number of such platforms have been applied to ancient DNA samples, the Illumina series are the dominant choice today, mainly because of high production capacities and short read production. Recently a potentially attractive alternative platform for palaeogenomic data generation has been developed, the BGISEQ-500, whose sequence output are comparable with the Illumina series. In this study, we modified the standard BGISEQ-500 library preparation specifically for use on degraded DNA, then directly compared the sequencing performance and data quality of the BGISEQ-500 to the Illumina HiSeq2500 platform on DNA extracted from 8 historic and ancient dog and wolf samples. The data generated were largely comparable between sequencing platforms, with no statistically significant difference observed for parameters including level (*P* = 0.371) and average sequence length (*P* = 0718) of endogenous nuclear DNA, sequence GC content (*P* = 0.311), double-stranded DNA damage rate (v. 0.309), and sequence clonality (*P* = 0.093). Small significant differences were found in single-strand DNA damage rate (δS; slightly lower for the BGISEQ-500, *P* = 0.011) and the background rate of difference from the reference genome (θ; slightly higher for BGISEQ-500, *P* = 0.012). This may result from the differences in amplification cycles used to polymerase chain reaction–amplify the libraries. A significant difference was also observed in the mitochondrial DNA percentages recovered (*P* = 0.018), although we believe this is likely a stochastic effect relating to the extremely low levels of mitochondria that were sequenced from 3 of the samples with overall very low levels of endogenous DNA. Although we acknowledge that our analyses were limited to animal material, our observations suggest that the BGISEQ-500 holds the potential to represent a valid and potentially valuable alternative platform for palaeogenomic data generation that is worthy of future exploration by those interested in the sequencing and analysis of degraded DNA.

## Background

As with many other disciplines, the advent of next-generation sequencing (NGS) platforms has revolutionized ancient DNA (aDNA) research. During the era of Sanger sequencing, the datasets within most studies were restricted to short lengths of mitochondrial DNA (mtDNA) or nuclear DNA (nuDNA), and at most, if one used multiplexing techniques, one could aim for mitochondrial genomes (mitogenomes) [1]. However, thanks to NGS techniques, with the right sample and sufficient funds, today practitioners are able to aim for relatively complete ancient nuclear genomes (hereafter referred to as palaeogenomes), even at the population level. While there are now a range of NGS technologies available to choose from, those favoured by the aDNA field are suited to the characteristically short DNA molecules that dominate aDNA extracts [2, 3]; thus long-read technologies such as the PacBio (Pacific Biosciences, CA, USA) and Minion (Oxford Nanopore Technologies, Oxford, UK) are not widely used. A range of technologies have been explored in the aDNA context, including the Roche/454 series [4–6], SOLID-4 [7], the now discontinued Helicos [8, 9], and the Ion Torrent series [10]. The undisputed workhorses, however, are the platforms within the Illumina series, principally due to a combination of factors that include sequencing cost per unit date, low sequencing error rate (ca 0.1% [11]), and simply the number of machines available upon which to sequence. Thus in recent years, the focus has been placed on the development and optimization of methods in order to increase data quality and reduce overall cost. Steps taken have included both tailoring library constructions and amplification methods toward the damaged endogenous aDNA, e.g., through exploiting blunt end ligations [12], removing steps associated with DNA loss [13], enzyme choice [14], or even focusing on direct ligation to single-stranded DNA [15], as well as improvement in the informatic tools that are used to process the Fastq data generated [16–19].

Today, therefore, Illumina-based sequencing has formed the basis of the overwhelming majority of palaeogenomic studies, including (but not limited to) draft genomes of humans [20] and related hominids [21–24], animals [9, 25–28], plants [29–31] and even pathogens [32–40], population genomic datasets [34, 41–46], metagenomic studies [47–50], and even insights into ancient transcriptomes [38, 51, 52] and epigenomes [53–57]. For recent reviews, see [58, 59].

Despite this remarkable progress, palaeogenomics still faces one significant limitation—the overall data generation cost. The per-base cost of Illumina-based NGS sequencing is falling thanks to improvements relating to flow-cell cluster density and the generation of longer reads (although for most aDNA this latter point is rarely beneficial). As such, today a modern human 3 gigabase haploid genome can be sequenced to ×30 coverage for as little as USD$1000 [60], and possibly even as low as USD$100 [61]. Palaeogenomicists, however, are not so fortunate, given that much (if not, in many cases, the majority) of the DNA in most ancient samples is derived from exogenous contaminants [4] such as microbes. While some methodological improvements such as optimized choice of tissue sources [62, 63], extraction methods [64–68], and various forms of enrichment help improve the endogenous DNA content [15, 56, 69–75], costs can still be many fold that for modern DNA data. Thus while attractive to many, the application of palaeogenomics has been largely restricted to the most well-funded research teams and spectacular research questions.

While Illumina has dominated the palaeogenomic sequencing market, in 2016, a new platform emerged that may offer considerable potential to the field—the combinatorial probe-anchor synthesis–based BGISEQ-500 [11]. The underlying technology combines DNA nanoball (DNB) nanoarrays [76] with polymerase-based stepwise sequencing, and its use has recently been validated as comparative in performance to the Illumina platforms when sequencing small noncoding RNAs [77] as well as resequencing modern human DNA [78]. The BGISEQ-500 has several features [11] that suggest it will be it attractive to aDNA users. First, its sequencing read-length capacity (currently up to either single read [SR] or paired end [PE] 100 bp) falls within lengths that are acceptable to most palaeogenomicists. Second, its high throughput—a single 2 channel flow cell can produce at least 500 million single-end reads per channel (thus up to at least 2 billion PE reads per flow cell) in only a few days. Third, at least the initial stages of the library construction method underlying the BGISEQ-500 are sufficiently close to the methods currently used for Illumina palaeogenomic sequencing, and thus can be easily modified based upon some of the abovementioned previous aDNA-related developments if needed. To fully explore this platform's potential for aDNA, we therefore undertook a direct performance comparison against Illumina technology by building libraries and sequencing 8 historic and ancient DNA extracts. To both keep the underlying variables as similar as possible and to exploit a recent (Illumina-based) methodological development that (i) simplifies library construction and minimizes hands-on time and economic cost [13] and (ii) performs at least as well as the Meyer and Kircher [12] blunt end ligation method that many palaeogenomicists favour, we did not use the original BGISEQ-500 library method, but rather developed a new protocol based on our recently developed blunt end single tube (BEST) method [13]. We subsequently undertook a range of bioinformatic analyses aimed at exploring whether the resulting sequence datasets (i.e., Illumina vs BGISEQ-500) exhibited significant differences with regards to a number of parameters that are currently believed important for aDNA studies.

## Data Description

DNA was extracted from 8 historic and ancient canid samples, chosen so as to represent a range of materials that are currently interesting to the palaeogenomics community (Table 1)—in particular with regards to the fragment sizes of the surviving DNA and the range of endogenous DNA content within them. Two of the samples are preserved hides of wolves (*Canis lupus*), between 91 and 148 years old, that are believed to contain relatively pure (free of enzymatic inhibitors), although heavily fragmented, DNA (a presumed side effect of the tanning process). The remaining samples are naturally preserved wolf, dog (*Canis familiaris*), or undetermined large canid remains dated between roughly 600 and 14 000 years old.

**Table 1:** Samples from which aDNA was extracted

Sample	Original ID	Material	Species	Locality	Age	Extraction
214	CN 214	Hide	Wolf	Uummannaq, Greenland	Before 1869 AD	A
1921	CN 1921	Hide	Wolf	Rosenvinge Bugt, Greenland	1925 AD	A
P84	MGUH VP 3332	Humerus	Wolf	Vølvedal, Greenland	ca. 7620 cal YBP	B
P83	NKA 1950 × 2906	Canine tooth	Dog	GUS, Greenland	ca. 600–1000 YBP	B
P79	ZMK 350/1982	Tibia	Dog	Qajâ, Greenland	ca. 3,6–2700 YBP	B
FRC	FRC	Cartilage	Large canid	Tumat, Siberia	ca. 14 122 cal YBP	C
L	L	Liver	Large canid	Tumat, Siberia	ca. 14 122 cal YBP	C
M1	M1	Muscle	Large canid	Tumat, Siberia	ca. 14 122 cal YBP	C

Sample CN 214 was acquired by and registered in the collections of the Natural History Museum of Denmark (NHMD) in 1869. According to museum records, the specimen was shot in Uummannaq, West Greenland, prior to 1869. CN 1921 is a wolf that was shot in Rosenvinge Bugt, East Greenland, in 1925, and then subsequently placed in the NHMD collections. MGUH VP 3332 is a find belonging to the Greenland National Museum (GNM), specifically a bone sample found on the surface 2 m above sea level in 1979 in Vølvedal Peary Land, North Greenland. The specimen has been directly dated to 6785 ± 100 ^14^C years BP (Ua-1346, calibrated age: 7620 years BP) [79]. NKA 1950 × 2906 is a tooth sample excavated at the Greenlandic Norse GUS site (Gården Under Sandet/The Farm Beneath the Sand) and is placed in the GNM collections. The site was settled by the Greenlandic Norse and inhabited between ca. 1000 to 600 years BP [80]. ZMK 350/1982 was excavated from the Saqqaq cultural Paleo-Eskimo site Qajâ and is placed in the GNM collections. Although the site in general has been dated to between 3600 and 2700 years BP, this particular sample is from the earliest occupation layers [81–83]. Last, samples FRC, L, and M1 are tissue samples from an extremely well preserved mummified canid found in the permafrost near the village Tumat in the Sakha Republic, Siberia, Russia. The specimen belongs to the collections of the Mammoth Museum in Yakutsk (Russia) and has been directly dated to 12 223 ± 34 ^14^C years BP (ETH-73412, calibrated age ranging from 12 297 BC to 12 047 years BC, with 95.4% likelihood calibrated to ca. 14 122 years BP); calibration was made using OxCal v. 4.2.4. [84].

Following DNA extraction, 2 aliquots of each extract were constructed in the Illumina and BGISEQ-500 libraries, respectively, using identical amounts of starting material (16.3 μl, ∼5–50 ng DNA input sample dependent), and then subsequently sequenced to enable bioinformatic comparisons on the data.

## Analyses and Discussion

We initially generated between 1.35 × 10^7^ and 5.94 × 10^7^ reads per Illumina library, and 2.32 × 10^7^ - 3.39 × 10^8^ reads per BGISEQ-500 library (Table 2; Supplemental Table S1). The dataset supporting the results of this article is available in the ERDA and GigaDB repositories (see Availability of supporting data). Following normalization of the data for read length and depth (Table 2), we found no statistically significant difference between the 2 datasets with regards to the % endogenous nuclear DNA and average length of endogenous DNA, several of the most important parameters for palaeogenomicists, given their fundamental role in affecting the overall financial cost of a study (Table 3). In contrast, there was a statistically significant platform-dependent difference in the % reads mapping to the mitochondrial genome, with fewer reads mapping in the BGISEQ-500 libraries. However, closer inspection of the data indicates that the total numbers of mtDNA reads are extremely low for 3 of the samples (Supplemental Table S1) and that for the remainder the numbers are extremely similar. As such, we do not believe there to be much significance behind this observation.

**Table 2:** Summary of data generated

Sample	Platform	Total reads	Normalized % reads retained after adapter removal	Normalized clonality	Normalized endogenous DNA (%)	Normalized length of uniquely mapped reads	θ	δD	δS	GC content (%)	mtDNA (%)
1921	Illumina	3.08E+07	94.69	0.11	58.73	40.77	0.008	0.008	0.154	51.58	4.51E-03
	BGISEQ-500	5.32E+07	83.97	0.15	59.37	42.14	0.009	0.008	0.132	50.42	2.57E-03
214	Illumina	1.35E+07	99.13	0.07	74.25	49.37	0.008	0.011	0.084	48.60	4.15E-03
	BGISEQ-500	1.98E+08	99.55	0.07	75.51	53.08	0.009	0.012	0.061	47.75	3.11E-04
FRC	Illumina	1.64E+07	99.54	0.03	11.58	73.05	0.008	0.012	0.399	44.01	4.55E-03
	BGISEQ-500	3.39E+08	99.79	0.02	10.22	75.63	0.012	0.012	0.325	43.64	1.98E-04
L	Illumina	2.91E+07	99.63	0.09	1.03	64.65	0.013	0.010	0.415	43.24	6.04E-03
	BGISEQ-500	2.44E+08	99.77	0.08	0.85	66.72	0.013	0.009	0.262	45.99	7.09E-04
M1	Illumina	5.10E+07	99.38	0.06	64.09	72.95	0.007	0.010	0.395	44.27	8.02E-03
	BGISEQ-500	1.79E+08	99.74	0.06	54.80	76.76	0.012	0.010	0.258	43.23	2.31E-03
P79	Illumina	4.18E+07	98.48	0.38	0.07	52.45	0.030	0.012	0.880	43.36	4.65E-06
	BGISEQ-500	8.55E+07	98.08	0.10	0.06	45.77	0.039	0.011	0.550	44.21	6.40E-07
P83	Illumina	2.77E+07	84.67	0.58	0.64	65.78	0.014	0.040	0.842	42.32	4.85E-04
	BGISEQ-500	2.32E+07	86.84	0.32	0.47	66.55	0.017	0.040	0.773	44.30	3.87E-04
P84	Illumina	5.94E+07	98.70	0.31	0.12	54.79	0.015	0.030	0.355	44.42	2.71E-06
	BGISEQ-500	1.57E+08	92.45	0.08	0.10	51.13	0.022	0.020	0.154	47.99	5.15E-07

**Table 3:** Results of statistical analyses of the data

Test	Paired *t*-test *t*	*P*-value
% reads retained	−1.131308	0.295
Clonality levels	−1.942886	0.093
% endogenous DNA	−0.956158	0.371
Endogenous DNA average read length	0.0375544	0.718
θ	3.366145	0.012^a^
δD	−1.09765	0.309
δS	−3.425669	0.011^a^
% GC	1.091076	0.311
% mtDNA	−3.073585	0.018^a^

^a^Significant at *P* < 0.05.

**Figure 1:**
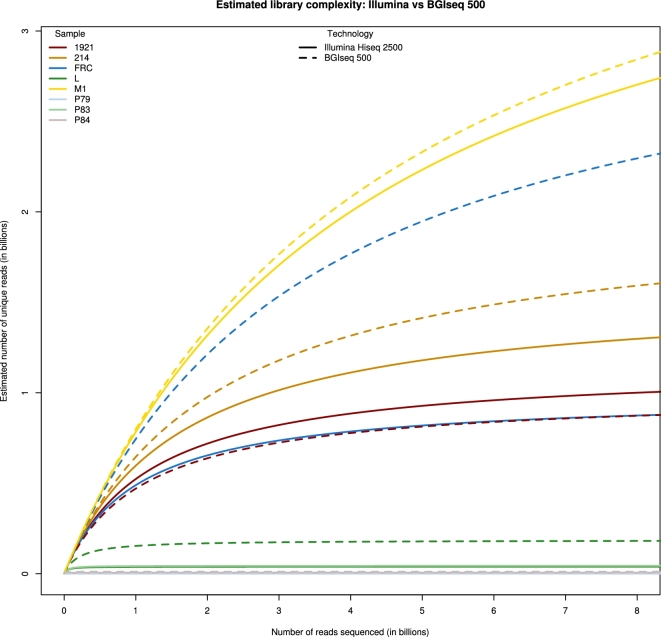
Library complexity estimated as the number of unique reads as a function of the total number of reads sequenced. These numbers are estimated and extrapolated using the program *preseq* [84]. The total number of reads sequenced for each library can be found in Table 2 and Supplemental Table S1. The solid lines are the estimates for the libraries sequenced on the Illumina HiSeq 2500 platform, while the dotted lines are the estimates for the libraries sequenced on the BGISEQ-500. Each of the 8 samples is represented by a different colour.

With regards to sequence accuracy, although double-strand (δD) sequence damage rates as estimated using MapDamage2.0 [17] showed no statistically significant difference, a small, yet statistically significant difference was observed for δS, the single-strand damage parameter (lower rate for BGISEQ-500) (Tables 2 and 3). Furthermore, we also observed a small, yet significant difference in the background rate of differences from the reference genome (MapDamage2.0 θ), with slightly higher values observed in the BGISEQ-500 platform (Tables 2 and 3). We hypothesize that both differences may be explained by the fact that, while the initial steps of the library build methodologies are similar, a greater number of polymerase chain reaction (PCR) cycles was used to amplify the Illumina libraries (Supplemental Table S3). This had a clear effect on overall library complexity as while there was no statistically significant difference with regards to library clonality levels or the % reads retained after initial filtering (Tables 2 and 3), when we used *preseq* [85] to extrapolate on the library complexity, we observed that in all but 1 case, the BGISEQ-500 platform provided richer libraries (Fig. 1). Alternatively, we hypothesize that an alternative explanation for the observed differences in δS and θ might relate to the relatively low genome coverage that we have for each sample. As such, each sample was sequenced over different parts of the genome, which in turn may lead to small biases in the error profiles. Ultimately, however, we feel that full resolution of the differences will require the generation of extensive extra data, and thus more will be learnt in future studies that use the BGISEQ-500.

**Figure 2:**
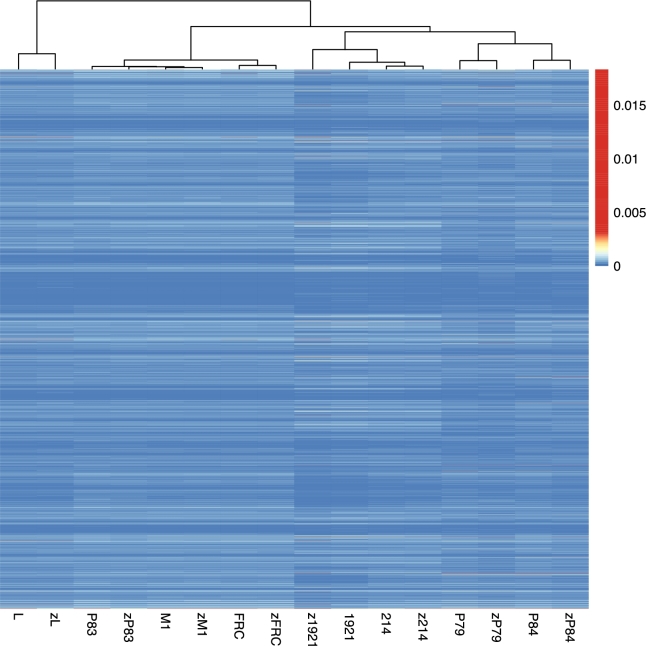
Heatmap of k-mer counts across libraries. Libraries (columns) were hierarchically clustered based on Pearson correlation. Proportion of each of the 4096 6-mer (rows) are depicted using colours.

We subsequently explored 2 further parameters that relate to whether there are method-specific biases with regards to which part of the genome is sequenced: k-mer frequency and GC content. k-mer content was consistent between methods for most of the samples, each sample pair clustered together. However, samples P83 and 1921 were exceptions to this pattern, with each method yielding slightly different k-mer distributions (Fig. 2). We note that the k-mer content of sample P83 is very similar to sample M1, which makes accurate clustering more challenging. The differences for sample 1921 are more difficult to explain, however, although 1 obvious point is that this is the sole BGISEQ-500 library to exhibit lower complexity than its Illumina pair, although it is not clear if/how this may affect the results.

**Figure 3:**
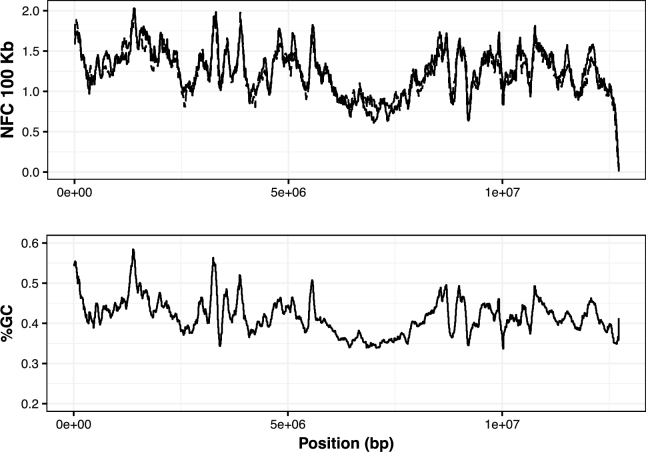
Top: median normalized fragment count (NFC) per 100 Kb windows, with 10 Kb offset for the sample 214 along scaffold_0. The solid line shows Illumina data, and the dotted line shows BGISEQ-500 data. Bottom: percentage GC calculated over the same the same windows as in the upper panel.

**Figure 4:**
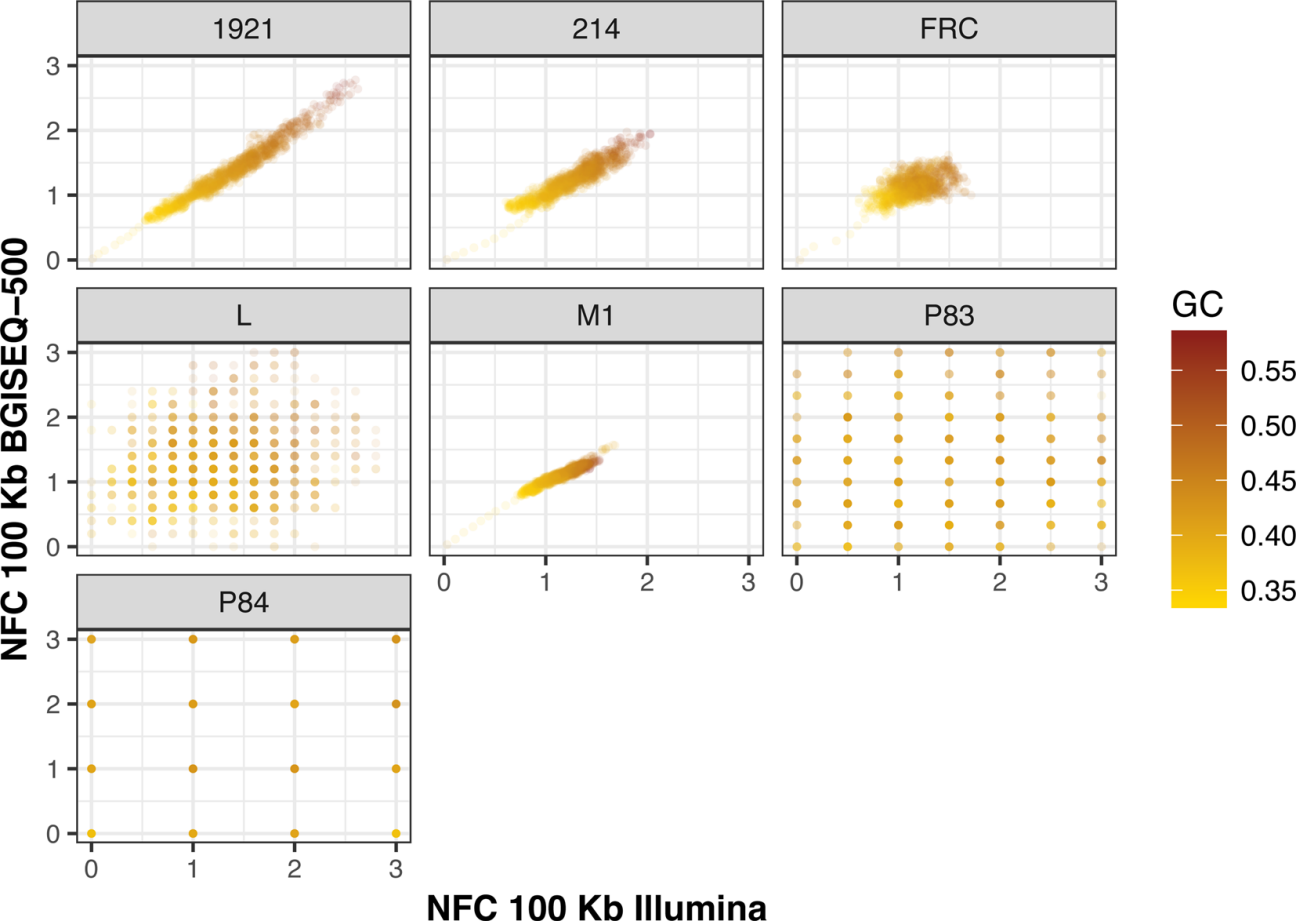
Median NFC of Illumina vs BGISEQ-500 for all samples in windows of 100 Kb, with an offset of 1 0Kb along scaffold_0. The color of each point corresponds to the windows’ GC content. For the high-quality samples (1921, 214, FRC, M1), a very good correlation of NFC between the 2 platforms can be observed. The fragment count seems to be correlated with GC content.

GC content was also largely consistent between methods. At a global level, we found no statistically significant difference in the average GC content (Tables 2 and 3), and in more refined analyses, we observed the fragment count for the same windows to be well correlated between BGISEQ-500- and Illumina-derived reads, both of which are correlated with GC content (Figs 3 and 4). We find high genome-wide coefficients of determination for samples 1921,214,L and M1, while these values are lower for samples FRC, P83, and P84 (see Table 4; the sample P79 was excluded from this analysis because of insufficient data). We believe these differences are most likely attributable to the overall endogenous DNA quality in the samples rather than the platforms’ technical performance as there is a trend of samples with lower endogenous DNA content having poorer correlations.

**Table 4:** Overview of *r*^2^ values for normalized fragment counts between Illumina and BGISEQ-500 for windows of 100 Kb

Sample	*r* ^2^ NFC whole genome
CN 1921	0.976
CN 214	0.965
FRC	0.772
L	0.904
M1	0.954
P83	0.084
P84	0.513

Our final analysis explored copy number variation (CNV) levels although, as mentioned above, the low genomic coverage of the data makes CNV analyses challenging. Nevertheless, the *r*^2^ values for the comparisons that pass our quality control range from 0.35 to 0.96 (Table 5). Furthermore, the observation of particular DNA extractions with excellent concordance values despite the nature of our experiment make it tempting to speculate that indeed both technologies are viable for high-quality CNV calls. For example, using 36-mers and accounting for all possible placements of a 36-mer, the sample M1 has a coverage of above ×1 on both platforms. Ultimately, however, it is not possible to discern from the present data whether the observed variation in CNV calls in the samples is due to differences in the sequencing platforms or the nature of the libraries; thus these results should be taken as preliminary, pending future validation.

**Table 5:** Coefficients of determination for copy number in the same genomic windows between platforms, for all extracts at varying resolution

	CW size					
Sample	1000 Kbp	100 Kbp	50 Kbp	10 Kbp	5 Kbp	1 Kbp
214	0.905^a^	0.331^a^	0.354^a^	0.506^a^	0.519^b^	0.433^c^
1921	0.963^a^	0.384^a^	0.392^a^	0.428^a^	0.432^b^	0.393^c^
FRC	0.582^a^	0.847^a^	0.870^a^	0.873^b^	0.870^c^	0.783^c^
L	0.941^b^	0.957^c^	0.964^c^	0.958^c^	0.955^c^	ND
M1	0.665^a^	0.943^a^	0.952^a^	0.953^a^	0.950^a^	0.910^b^
P79	0.672^b^	ND	ND	ND	ND	ND
P83	0.203^b^	0.003^c^	0.004^c^	0.003^c^	0.002^c^	ND
P84	0.919^b^	0.001^c^	0.001^c^	ND	ND	ND

ND = insufficient data for at least 1 platform.

^a^Denotes a pass of quality control (visual inspection of read depth density in control regions and proper SW/CW and LW/CW ratios).

^b^Denotes suboptimal quality, e.g., not perfectly symmetrical, bell-shaped read depth distribution in control regions.

^c^Denotes failed QC for at least 1 platform.

## Potential Implications

Our study represents the first exploration of the applicability of the BGISEQ-500 as an alternative sequencing platform to the Illumina series for palaeogenomic sequencing, and in doing so we present a library build protocol to generate such data. Although our study is based on only 8 specimens, given that their ranges of endogenous DNA content (<1–75%) and normalized average endogenous DNA sequence lengths (ca 42–76 bp) are typical of many other ancient samples, we anticipate that our results will be indicative of the platform on such material in general. Overall, the results are extremely promising—the BGISEQ-500’s performance is comparable over all parameters tested, with the exception of the very slightly elevated error rate observed (although in contrast we observe higher library complexity and lower δS; thus overall we feel this will not represent a major concern to palaeogenomic studies). We do caution, however, that due to the small size of the dataset (both sample numbers and sequencing depth), at this point we are not able to offer any comment as to how this overall evidence of consistency may translate into downstream analyses involving whole genome summary statistics. Thus we strongly advocate that those who may be interested in using the BGISEQ-500 platform in genomic population data explore this point further. Furthermore, as additional datasets are generated, we look forward to the results of analyses that might wish to compare the relative performance of different sequence alignment and variant calling software on such data. Ultimately, however, we anticipate that our findings will stimulate considerable interest in the BGISEQ-500 platform by palaeogenomic research teams attempting to reconstruct ancient genomes and transcriptomes, and we look forward to future exploration of its potential across a wider range of ancient substrates.

## Methods

### DNA extraction

DNA was extracted using 1 of 3 different methods (designated A, B, C) (Table 1), as deemed appropriate for the choice of tissue. Methods A and C involved digestion in a proteinase K–containing buffer, following Gilbert et al. [62], while method B involved digestion in a proteinase K–urea buffer, following Ersmark et al. [86]. All samples were predigested at 56°C for 1 hour, after which the buffer was changed and then a second 12-hour digest was performed. Digests from method A used organic solvents (phenol: chloroform) and Qiagen MinElute columns (Qiagen, Hilden, Germany), following Carøe et al. [13]. Digests from methods B and C were centrifuged at 6000 ×G for 1 minute, after which 500 μl supernatant was mixed 1:8 with a binding buffer as detailed in Allentoft et al. [42], then centrifuged through Monarch DNA Cleanup Columns (New England Biolabs, MA, USA). DNA bound to the columns was washed with 800 μl buffer PE (Qiagen), then eluted using 2 washes in 17 μl buffer EB (Qiagen)—each with an incubation for 5 minutes at 37°C. Prior to library construction, small aliquots of each extract were analysed on an Agilent 2200 TapeStation HS chip (Agilent Technologies, Palo Alto, CA, USA) for fragment size estimation and molar concentration.

### Library construction

Two aliquots of each extract were constructed in the Illumina and BGISEQ-500 libraries, respectively, using identical amounts of starting material (16.3 μl, ∼5–50 ng DNA input sample dependent) (Supplemental Table S2). Library blanks and index PCR blanks were also included to evaluate the potential contaminations during the library building process. Illumina libraries were constructed using a method based on the recently published single tube “BEST” protocol, largely following Carøe et al. [13], although with some modifications (Supplemental File F1). To enable direct comparison of the sequencing methods, we chose not to use the conventional BGISEQ-500 library construction protocol. Rather, given the similarities between the initial processes of library construction between both methods (DNA end repair and adapter ligation), we modified the BEST protocol to be BGISEQ-500 compatible. Specifically, the standard Illumina-compatible adapters were replaced with BGISEQ-500-compatible adapters AD1 and AD2 (Supplemental Table S4). These adapters were synthesized as 2 pairs of complementary oligonucleotides (AD1_Long and AD1_Short, and AD2_Long and AD2_Short, respectively), then prepared into the final adapters, AD1 and AD2. Specifically, adapters were first diluted to 500 μM with ×1 TE buffer (10 mM Tris-HCl, 1 mM EDTA, pH 8.0, Sigma-Aldrich). Subsequently, an equimolar concentration of each pair of long and short adapters was mixed together and hybridized through incubation at 95°C for 1 minute, followed by a decrease in temperature with 0.1°C/s from 95°C to 12°C. After hybridization, adapters AD1 and AD2 were mixed and diluted at a concentration of 10 μM prior to their use in the library construction. We additionally designed BGISEQ-500-compatible library amplification primers for use in the library amplification steps that included 8 alternate sequencing indices in the reverse primers (Supplemental Table S4).

Following the final Bst fill-in step during library build, all libraries were mixed with 1:5 volume of PB binding buffer (Qiagen) and purified using Monarch® DNA Cleanup Columns, then washed with 750 μl buffer PE (Qiagen) and eluted in 40 μl buffer EB (Qiagen) after a 5-minute incubation at 37°C.

#### Illumina library PCR amplification and sequencing

Quantitative real-time PCR (qPCR) was used to estimate the required number of cycles during library index PCR. Each qPCR was performed in a 20 μl reaction volume using 1:20 dilution of purified library template, 0.2 mM dNTPs (Invitrogen), 0.04 U/μl AmpliTaq Gold DNA polymerase (Applied Biosystems, Foster City, CA, USA), 2.5 mM MgCl_2_ (Applied Biosystems), 1X GeneAmp® 10X PCR Buffer II (Applied Biosystems), 1 μl SYBR Green (Invitrogen, Carlsbad, CA, USA), 0.2 μM forward and reverse primers mixture (IS7 and IS8 primers [12]), and 13.48 μl AccuGene molecular biology water (Lonza). qPCR cycling conditions were 95°C for 10 minutes, followed by 40 cycles of 95°C for 30 seconds, 60°C for 60 seconds, and 72°C for 60 seconds using the MX3005 qPCR machine (Agilent).

Post-qPCR, library index amplifications were performed in 100 μl PCR reactions that contained 20 μl of purified library, 0.2 mM dNTPs (Invitrogen), 0.1 U/μl AmpliTaq Gold DNA polymerase (Applied Biosystems), 2.5 mM MgCl_2_ (Applied Biosystems), 1X GeneAmp® 10X PCR Buffer II (Applied Biosystems), 0.4 mg/ml BSA (New England Biolabs Inc), 0.2 μM of each forward (Illumina InPE 1.0 forward) and custom made reverse primers, and 51.2 μl AccuGene molecular biology water (Lonza, Basel, CH). PCR cycling conditions were: initial denaturation at 95°C for 12 minutes followed by 13 to 21 cycles of 95°C for 30 seconds, 60°C for 30 seconds, and 72°C for 40 seconds, and a final elongation step at 72°C for 5 minutes. Post-PCR, libraries were purified with QiaQuick columns (Qiagen) and eluted with 30 μl buffer EB (Qiagen) after an incubation for 10 minutes at 37°C. Small aliquots of this purified product were used for quantification and fragment size estimation using the High-Sensitivity DNA Assay for the Bioanalyzer 2100 (Agilent). Subsequently, there was a final purification using the AMpure XP system (Agentcourt, Beckman Counter, Indianapolis, IN, USA) with ×1.8 beads:library ratio, in order to remove any persisting primer dimers or other molecules with a fragment size of <100 bp. Last, libraries were pooled in equimolar concentrations (∼9.4 nM) and sequenced on the Illumina HiSeq platform in 80 bp single read mode by The Danish National High-Throughput DNA Sequencing Centre.

#### BGISEQ-500 library PCR amplification

Initial processing steps for the purified BGISEQ-500 libraries were largely similar to those used in the Illumina libraries, although with the following modifications. First, the libraries were qPCR-quantified using the *CommonprimerBGI forward* primer and 1 of the indexed reverse primers (Supplemental Table S4). Second, subsequent index PCR amplifications used 8 to 15 cycles (Supplemental Table S3) with *CommonprimerBGI forward* primer and the indexed reverse primers (Supplemental Table S4). Third, because several of the BGISEQ-500 libraries exhibited residual adapter dimers after the initial purification post-index PCR, each purified BGISEQ-500 library was split to 2 aliquots (∼12.5 μl each), and 1 of each aliquot was subject to an extra purification to remove any residual primer dimers (Supplemental Table S2). Each of these aliquots was sequenced independently. We note that several of the extrapurified libraries showed small improvements with regards to overall adapter dimer content in the generated sequence (Supplemental Table S5), and our initial impression is that this extra purification step may be worth undertaking if high levels of adapter dimers are found post-index PCR.

#### BGISEQ-500 library circularization and sequencing

All amplified libraries were subsequently sent to BGI for circularization and sequencing on the BGISEQ-500 platform. For circularization, PCR products with different barcodes were pooled together at equimolar concentration to yield a final amount of 80 ng. Pools contained both the samples relevant to this study as well as those from other projects (Supplemental Table S6). Each pool was subsequently heat-denatured, and the single-strand DNA was mixed with MGIEasyTM DNA Library Prep Kit V1 (PN:85–05533-00, BGI, Shenzhen, China), containing 5 μl splint oligo, 6 μl splint Buffer, 0.6 μl ligation Enhancer, and 0.2 μl ligation (Enzyme and NF water) to form a 60 μl reaction system, which was subsequently incubated at 37°C for 30 minutes. Last, 20 μl of each single-circle-library pool was used as input to prepare the DNB. Each pool was then sequenced on 1 lane, using 100SR chemistry with BGISEQ-500RS High-throughput sequencing kit (PN: 85–05238-01, BGI). Postsequencing, the data were automatically demultiplexed by index.

### Data analyses

The raw reads obtained from the HiSeq 2500 and BGISEQ-500 were analysed using FastQC (FastQC, RRID:SCR_014583) [87] to compute the quality metrics of the reads, such as base sequence qualities, base sequence content, %GC, and sequence composition. With the exception of the analysis on the standard vs extrapurified BGISEQ-500 libraries (Supplemental Table S5), both BGISEQ-500 libraries from each extract were treated as a single dataset. We also compared the quality metrics of the reads from the same samples across the 2 platforms to ensure that the sequencing platform did not have a large impact on the quality metrics of the reads.

Once the read qualities were verified using FastQC (FastQC, RRID:SCR_014583), we used the PALEOMIX pipeline (PALEOMIX, RRID:SCR_015057) [18] to trim the adapter sequences, trim Ns and low-quality bases from the ends of reads, estimate ancient DNA damage, and finally map the trimmed reads to the reference genome. The individual steps of the pipeline are detailed below. We highlight that the values presented in Table 2 are normalized to account for sequencing read depth and length, while Supplemental Table S1 contains both the original and the normalized values.

#### Adapter removal and trimming

The first step of the initial processing of the reads involved trimming the adapter sequences from the ends of the reads. Since the samples consist of degraded DNA, many of the sequenced reads contain the platform-specific adapters at the 3^΄^ end of the reads. AdapterRemoval (v. 2.1.3) [88] was used to trim the adapter sequences from the ends of the reads using the default mismatch rate of 1/3. In addition, bases with a quality score of less than 2 and unidentified bases (Ns) at the ends of reads were trimmed. Finally, only reads that were longer than 25 bases were retained for downstream analyses.

#### Mapping, indel realignment, and duplicate removal

The trimmed reads were mapped to the wolf reference genome [89] using the mem algorithm in bwa (BWA, RRID:SCR_010910; v. 0.7.10), using the default settings for the mapping algorithm. The mapped reads were subsequently processed using the GATK (v. 3.3.0) indel realigner (GATK, RRID:SCR_001876) [90, 91] to fix the alignment issues arising from the presence of short indels at the beginnings and ends of reads. Since there are no catalogs of indel variations in the species included in this study, the realignment step was done using a set of indels within each sample. After the indel realignment step, the PCR duplicates were removed from the alignments using the MarkDuplicates program from Picard tools (Picard, RRID:SCR_006525; v. 1.128) [92].

#### DNA damage patterns

The DNA damage patterns and parameters were estimated using mapDamage (v.2.0.6; mapDamage, RRID:SCR_001240) [17] using a subsample of 100 000 reads from the set of mapped reads. The 3 main parameters estimated using mapDamage were θ, δD, and δS. δD and δS estimate the probability of cytosine deamination (driven by hydrolytic DNA damage) in a double- and single-stranded context, while θ estimates the background rate of difference between the reference and sample after accounting for DNA damage. Using these estimated parameters, the base qualities of putatively damaged bases were recalibrated to a lower score. The program was also used to compute the relative abundance of C→T changes at the 3^΄^ ends and A→G changes at the 5^΄^ ends of the reads and compare them across the 2 platforms.

#### Clonality, endogenous DNA content, and library complexity estimation

The clonality of each library was computed from the reads that were identified by the MarkDuplicates program during the duplicate identification and removal step. The clonality was computed as the ratio of the number of reads retained after duplicate removal and the number of reads retained after the adapter removal and trimming step. The endogenous content of the library was computed as the ratio of the number of reads mapping uniquely to the reference genome and the number of reads retained after adapter removal. Note that this is 1 possible definition of the endogenous content, here defined as the proportion of usable reads obtained from a library, and the numbers given in Table 2 and Supplemental Table S1 will allow you to compute the values for other definitions of endogenous content.

The complexity of each library was estimated and extrapolated using the library complexity extrapolation model in the program *preseq* [85], which uses a nonparametric Bayesian Poisson model to estimate the gain in number of unique fragments when the library is sequenced deeper. Instead of using the aligned reads to estimate the library complexity, we used the counts of the number of duplicates in the bams generated by paleomix as input to *preseq*. The library complexity was estimated up to a maximum of a total of 10 billion reads sequenced per library.

#### Mapping to the wolf mitochondrial genome

Since the draft de novo wolf genome does not contain information on scaffolds that are annotated as belonging to the mitochondria, we could not identify reads that mapped to the mitochondrial genome using the initial set of mapped reads. To overcome this problem, we downloaded a complete mitochondrial genome from NCBI (GenBank Accession: AM711902) [93] and mapped the adapter trimmed reads to this complete mitochondrial genome. The same steps, including indel realignment and DNA damage–related recalibration of quality scores, were performed for the reads aligned to the mitochondria.

#### K-mer frequency

To compare the sequence content of the reads obtained from the 2 sequencing platforms, we computed the k-mer frequencies in the reads from the same sample using the 2 technologies. Since the raw reads are enriched in adapter sequences and do not accurately reflect the sequence content of the underlying endogenous DNA molecules in the library, we restricted the k-mer analysis to reads that mapped to the genome after going through both adapter trimming and duplicate read removal. For each library, we sampled 100 000 reads from the reads mapped to the reference genome using samtools (v. 1.2; SAMTOOLS, RRID:SCR_002105) [94, 95] and seqtk (v. 1.0) [96]. From these subsampled reads, we computed the 6-mer frequencies using jellyfish (Jellyfish, RRID:SCR_005491) [97].

#### Relative abundance vs GC content

The relationship between read abundance in a given genomic region and its GC content is well known and characterized for the Illumina platform [98]. For methods that depend upon depth of coverage or fragment count, such as measuring absolute copy number or expression levels, this bias needs to be taken into consideration and corrected for; otherwise, its magnitude might confound the signal in question. We therefore compared the GC content of the mapped endogenous DNA for the 2 platforms in several ways. First, the basic GC percentage was calculated from all endogenous reads. Second, we partitioned the reference genome into bins of 100 Kbps, with an offset of 10 Kbps, and calculated the GC percentage of each bin. We then mapped all datasets onto the reference and counted the number of mapped fragments in each bin. To account for differences in sequencing depth, we randomly subsampled mapped reads from the platform with the higher coverage to an equal amount of mapped bases of the platform with lower coverage, and then normalized the number of mappings by the median number of mappings for each extract.

#### CNV on low-coverage data

Fluctuations in depth of sequencing coverage can be used to generate personal genome-wide copy number maps of an individual as read depth is known to strongly correlate with copy number for several platforms [99]. We sought to assess whether the same techniques might be applied to data generated on the BGISEQ-500. To this end, we generated individual genome-wide CN maps of all extracts and both platforms in varying window sizes, from 1 Kbp to 1 Mbp, to account for fluctuation in coverage, and checked concordance between them. It is worth noting that using ancient DNA libraries poses a particular challenge to this assessment as some inherent characteristics of this type of data (such as unequal degradation, fragmentation, or clonality during library preparation) make it difficult to pinpoint the source of variability between 2 call sets for a given extract, given a lack of concordance. Specifically, low effective coverage and poor DNA quality make high-resolution maps not feasible for many of the libraries used in this part of the project.

We masked out any repeats in the reference assembly, as identified by both *repeat masker* (RepeatMasker, RRID:SCR_012954) [100] and tandem *repeat finder* [101]. Additionally, to identify repeats that have been potentially missed by the aforementioned algorithms, we chopped up the masked assembly into 36-mers with an offset of 5 bp. These were then mapped back onto the assembly using GEM (GEM, RRID:SCR_005339) [102] with a maximum divergence set to 95% and retaining all possible mappings. All 36-mers with more than 20 placements along the genome were additionally masked out. We then generated nonoverlapping 36-mers from the production reads and mapped them onto the extensively masked reference assembly using GEM, allowing for a maximum divergence of 95% and retaining all possible placements. To call absolute copy number, the reference was portioned in windows of 1, 5, 10, 50, 100, and 1000 Kbps of nonoverlapping, nonrepetitive sequences with *mrCanavar* (mrCaNaVaR, RRID:SCR_003135) [99], meaning that the genomic coordinates of the windows may span more than the window size if repeats are present within it. Importantly, as reads may not properly map at the boundaries of maskings, we introduced an additional padding of 36 bp. We then iteratively excluded all windows that represent outliers with respect to a normal distribution to identify a set of “control regions.” After correcting for GC content, the median depth of coverage in these control regions was used to normalize all windows and thus assign an absolute copy number to them. The concordance was calculated as the coefficient of determination of a linear model over corresponding to windows of the same extract between the 2 platforms. Additional quality control involved visually inspecting the normalized read depth distribution of the aforementioned control regions. In a good sample, this should be a symmetrical, bell-shaped curve centered at 2. We visually inspected all distributions and classified them as good, neutral, or bad, based on shape and symmetry. In addition to the aforementioned windows (called copy windows [CWs]), we also calculated normalized read depths the same size as CWs in terms of nonrepetitive sequence, with a fixed offset of the window size, but including repetitive sequence (called short windows [SWs]), and windows 5 times the size of a copy window (called long windows [LWs]), with an offset of 5 times the size of a copy window, but including repetitive sequence. As an additional quality control, the ratios of read depth of SW/CW should be around 1, and the ratios of read depth of LW/CW around 5, given proper sampling of the genome.

## Additional files

Supplemental File F1: Improvements to original BEST library building protocol (see additional file).

Supplemental Table S1: Full sequence data information (see additional file).

Supplemental Table S2: Sequence library identifiers.

Supplemental Table S3: The number of index PCR cycles used in each sample.

Supplemental Table S4: The sequences of BGISEQ-500 adapters and index primers used in this study.

Supplemental Table S5: Adapter dimer content of initial and extrapurified BGISEQ-500 libraries.

Supplemental Table S6: Library pooling for BGISEQ-500 library circularization reactions.

## Abbreviations

δD: MapDamage 2.0 double-strand DNA damage rate; δS: MapDamage 2.0 single-strand DNA damage rate; θ: MapDamage 2.0 DNA damage-corrected error rate; aDNA: ancient DNA; BEST: blunt end single tube; CN: copy number; CNV: copy number variation; CW: copy window; DNB: DNA nanoball; GNM: Greenland National Museum; LW: long window; NFC: normalized fragment count; NGS: next-generation sequencing; NHMD: Natural History Museum of Denmark; PE: paired end; SR: single read; SW: short window; YBP: years before present.

## Supplementary Material

GIGA-D-17-00050_Original-Submission.pdf

GIGA-D-17-00050_Revision-1.pdf

Response-to-Reviewer-Comments_Original-Submission.pdf

Reviewer-1-Report-(Original-Submission).pdf

Reviewer-2-Report-(Original-Submission).pdf

Reviewer-3-Report-(Original-Submission).pdf

Reviewer-3-Report-(Revision-1).pdf

Mak_Supplemental_File_F1.docx

Mak_TableS1.xlsx

Mak_Tables_S2-S6.pdf

## Availability of supporting data

Raw sequencing data are available from the SRA [PRJEB21089]. All other datasets supporting the results of this article are available in the ERDA repository [103] and *GigaScience* repository, GigaDB [104]. DNA extraction and library construction protocols presented here are also archived in protocols.io [105].
