# Erratum to: Comparative performance of the BGISEQ-500 vs Illumina HiSeq2500 sequencing platforms for palaeogenomic sequencing

**DOI:** 10.1093/gigascience/giy151

**Published:** 2018-12-07

**Authors:** Sarah Siu Tze Mak, Shyam Gopalakrishnan, Christian Carøe, Chunyu Geng, Shanlin Liu, Mikkel-Holger S Sinding, Lukas F K Kuderna, Wenwei Zhang, Shujin Fu, Filipe G Vieira, Mietje Germonpré, Hervé Bocherens, Sergey Fedorov, Bent Petersen, Thomas Sicheritz-Pontén, Tomas Marques-Bonet, Guojie Zhang, Hui Jiang, M Thomas P Gilbert

**Affiliations:** 1 Centre for GeoGenetics, Natural History Museum of Denmark, University of Copenhagen, Øster Voldgade 5–7,1350 Copenhagen, Denmark; 2 DTU Bioinformatics, Department of Bio and Health Informatics, Technical University of Denmark, Building 208, DK-2800 Lyngby, Denmark; 3 BGI-Shenzhen, Shenzhen 518083, China; 4 China National GeneBank, BGI-Shenzhen, Shenzhen 518083, China; 5 Natural History Museum, University of Oslo, PO Box 1172 Blindern, N-0318 Oslo, Norway; 6 The Qimmeq Project, University of Greenland, Manutooq 1, PO Box 1061, 3905 Nuussuaq, Greenland; 7 Institute of Evolutionary Biology (UPF-CSIC), PRBB, Dr. Aiguader 88, 08003 Barcelona, Spain; 8 CNAG-CRG, Centre for Genomic Regulation (CRG), Barcelona Institute of Science and Technology (BIST), Baldiri i Reixac 4, 08028 Barcelona, Spain; 9 OD Earth and History of Life, Royal Belgian Institute of Natural Sciences, Vautierstraat 29, 1000 Brussels, Belgium; 10 Department of Geosciences, Palaeobiology, University of Tübingen, Tübingen, Germany; 11 Senckenberg Centre for Human Evolution and Palaeoenvironment, University of Tübingen, Tübingen, Germany; 12 Mammoth Museum, Institute of Applied Ecology of the North of the North-Eastern Federal University, ul. Kulakovskogo 48, 677980 Yakutsk, Russia; 13 Catalan Institution of Research and Advanced Studies (ICREA), Passeig de Lluís Companys, 23, 08010, Barcelona, Spain; 14 Centre for Social Evolution, Department of Biology, Universitetsparken 15, University of Copenhagen, Copenhagen DK-2100, Denmark; 15 Trace and Environmental DNA Laboratory, Department of Environment and Agriculture, Curtin University, 6102 Perth, Australia; 16 Norwegian University of Science and Technology, University Museum, 7491 Trondheim, Norway

**Correction**

In the article, ‘Comparative performance of the BGISEQ-500 vs Illumina HiSeq2500 sequencing platforms for palaeogenomic sequencing, GigaScience 2017 6:8’ by Mak *et al*. [1], the first author's names appear incorrectly in References 104 and 105.

The correct names in References 104 and 105 are:

104. Mak SST, Gopalakrishnan S, Carøe C et al. Supporting data for ‘Comparative performance of the BGISEQ-500 vs Illumina sequencing platforms for palaeogenomic sequencing.’ GigaScience Database 2017. http://dx.doi.org/10.5524/100303 (accessed 1 March 2017)

105. Mak SST, Gopalakrishnan S, Carøe C et al. Protocols from ‘Comparative performance of the BGISEQ-500 vs Illumina sequencing platforms for palaeogenomic sequencing.’ protocols.io 2017. http://dx.doi.org/10.17504/protocols.io.h99b996 (accessed 1 March 2017)

There is an error in numbering of Supplemental Table S6 in Supplemental File F1. The correct numbering should be Supplemental Table S7 and its corresponding text in the last sentence of first paragraph in Supplemental File F1 should be: Supplemental Table S7 gives an overview of the different conditions, amount of enzyme and addition of Reaction Enhancer.
