# Supplementary material for: Comparative performance of the BGISEQ-500 vs Illumina HiSeq2500 sequencing platforms for palaeogenomic sequencing
Source: Gigascience. 2017 Jun 26;6(8):1–13. doi: 10.1093/gigascience/gix049 (PMC5570000; doi:10.1093/gigascience/gix049)
Supplement: GIGA-D-17-00050_Original-Submission.pdf [file gix049_giga-d-17-00050_original-submission.pdf]

GigaScience

Comparative performance of the BGISEQ-500 vs Illumina sequencing platforms for palaeogenomic sequencing

--Manuscript Draft--

**Manuscript Number:**	GIGA-D-17-00050
**Full Title:**	Comparative performance of the BGISEQ-500 vs Illumina sequencing platforms for palaeogenomic sequencing
**Article Type:**	Research
**Funding Information:**	European Research Council (681396) — Prof Marcus Thomas Pius Gilbert<br>H2020 Marie Skłodowska-Curie Actions (H2020-MSCA-ETN-643063) — Prof Marcus Thomas Pius Gilbert<br>Teknologi og Produktion, Det Frie Forskningsråd (4005-00107) — Prof Marcus Thomas Pius Gilbert<br>China National Genebank — Dr Hui Jiang<br>BGI Shenzhen — Dr Hui Jiang<br>FEDER (BFU2014-55090-P) — Mr Lukas Kuderna, Dr Tomas Marques-Bonet<br>Secretaria d'Universitats i Recerca del Departament d'Economia i Coneixement de la Generalitat de Catalunya — Dr Tomas Marques-Bonet

Funder	Recipient
European Research Council (681396)	Prof Marcus Thomas Pius Gilbert
H2020 Marie Skłodowska-Curie Actions (H2020-MSCA-ETN-643063)	Prof Marcus Thomas Pius Gilbert
Teknologi og Produktion, Det Frie Forskningsråd (4005-00107)	Prof Marcus Thomas Pius Gilbert
China National Genebank	Dr Hui Jiang
BGI Shenzhen	Dr Hui Jiang
FEDER (BFU2014-55090-P)	Mr Lukas Kuderna<br>Dr Tomas Marques-Bonet
Secretaria d'Universitats i Recerca del Departament d'Economia i Coneixement de la Generalitat de Catalunya	Dr Tomas Marques-Bonet

Abstract:

Background: Ancient DNA research has been revolutionised following development of 'Next Generation' Sequencing platforms. Although a number of such platforms have been applied to ancient DNA samples, the Illumina series are the dominant choice today, mainly because of high production capacities and short read production. Recently a potentially attractive alternative platform for palaeogenomic data generation has been developed, the BGISEQ-500, whose sequence output are comparable with the Illumina series. In this study, we modified the standard BGISEQ-500 library preparation specifically for use on degraded DNA, then directly compared the sequencing performance and data quality of the BGISEQ-500 to the Illumina HiSeq2500 platform, on DNA extracted from eight historic and ancient dog and wolf samples.
Results: The data generated was largely comparable between sequencing platforms, with no statistically significant difference observed for parameters including level and average sequence length of endogenous nuclear DNA, sequence GC content, double stranded DNA damage rate, and sequence clonality. Small significant differences were found in single strand DNA damage rate (δS, slight lower for the BGISEQ-500) and the background rate of difference from the reference genome (θ, slightly higher for BGISEQ-500). This may result from the differences in amplification cycles used to PCR amplify the libraries. A significant difference was also observed in the level of endogenous mitochondrial DNA sequenced, although we believe this is likely an artifact of the very low level of endogenous material in three of the samples.
Conclusions: Although we acknowledge our analyses were limited to animal material, our observations suggest that the BGISEQ-500 holds the potential to represent valid and potentially valuable alternative platform for palaeogenomic data generation, that is worthy of future exploration by those interested in the sequencing and analysis of degraded DNA.

**Corresponding Author:**	Marcus Thomas Pius Gilbert<br>University of Copenhagen<br>DENMARK
**Corresponding Author Secondary Information:**	
**Corresponding Author's Institution:**	University of Copenhagen
**Corresponding Author's Secondary**	

Powered by Editorial Manager® and ProduXion Manager® from Aries Systems Corporation

**Institution:**	
**First Author:**	Sarah Mak
**First Author Secondary Information:**	
**Order of Authors:**	Sarah Mak
	Shyam Gopalakrishnan
	Christian Carøe
	Chunyu Geng
	Shanlin Liu
	Mikkel Sinding
	Lukas Kuderna
	Wenwei Zhang
	Shujin Fu
	Filipe Vieira
	Bent Petersen
	Thomas Sicheritz-Ponten
	Tomas Marques-Bonet
	Guojie Zhang
	Hui Jiang
	Marcus Thomas Pius Gilbert
**Order of Authors Secondary Information:**	
**Opposed Reviewers:**	Mattias Meyer<br>Max Plank Evolutionary Anthropology, Leipzig<br><br>Direct competitor on ancient method development. We request you exclude anyone from this institute from reviewing the manuscript
	Johannes Krause<br>Max Planck Science of Human History, Jena<br><br>Direct competitor, we request you exclude any reviewer from this institute.
**Additional Information:**	
**Question**	**Response**
Are you submitting this manuscript to a special series or article collection?	No
**Experimental design and statistics**<br><br>Full details of the experimental design and statistical methods used should be given in the Methods section, as detailed in our Minimum Standards Reporting Checklist. Information essential to interpreting the data presented should be made available in the figure legends.<br><br>Have you included all the information requested in your manuscript?	Yes

**Resources**<br><br>A description of all resources used, including antibodies, cell lines, animals and software tools, with enough information to allow them to be uniquely identified, should be included in the Methods section. Authors are strongly encouraged to cite Research Resource Identifiers (RRIDs) for antibodies, model organisms and tools, where possible.<br><br>Have you included the information requested as detailed in our Minimum Standards Reporting Checklist?	Yes
**Availability of data and materials**<br><br>All datasets and code on which the conclusions of the paper rely must be either included in your submission or deposited in publicly available repositories (where available and ethically appropriate), referencing such data using a unique identifier in the references and in the "Availability of Data and Materials" section of your manuscript.<br><br>Have you have met the above requirement as detailed in our Minimum Standards Reporting Checklist?	Yes

Powered by Editorial Manager® and ProduXion Manager® from Aries Systems Corporation

Manuscript Click here to download Manuscript Mak_et_al_master_v5.docx

Comparative performance of the BGISEQ-500 versus Illumina HiSeq2500 sequencing platforms for palaeogenomic sequencing

Sarah Siu Tze Mak[1a†], Shyam Gopalakrishnan[1b†], Christian Carøe[1,2c†], Chunyu Geng[3d†], Shanlin Liu[1,4e], Mikkel-Holger S. Sinding[1,5,6f], Lukas F.K. Kuderna[7,8g], Wenwei Zhang[3h], Shujin Fu[3i], Filipe G. Vieira[1j], Bent Petersen[2k], Thomas Sicheritz-Ponten[2l], Tomas Marques-Bonet[7-9m], Guojie Zhang[4,10n], Hui Jiang[3o*], M. Thomas P. Gilbert[1,11,12p*]

[1]Centre for GeoGenetics, Natural History Museum of Denmark, University of Copenhagen, Øster Voldgade 5-7, 1350 Copenhagen, Denmark

[2]DTU Bioinformatics, Department of Bio and Health Informatics, Technical University of Denmark, Building 208, DK-2800 Lyngby, Denmark

[3]BGI-Shenzhen, Shenzhen 518083, China

[4]China National GeneBank, BGI-Shenzhen, Shenzhen 518083, China

[5]Natural History Museum, University of Oslo, PO Box 1172 Blindern, N-0318 Oslo, Norway

[6]University of Greenland

[7]Institute of Evolutionary Biology (UPF-CSIC), PRBB, Dr. Aiguader 88, 08003 Barcelona, Spain.

[8]CNAG-CRG, Centre for Genomic Regulation (CRG), Barcelona Institute of Science and Technology (BIST), Baldiri i Reixac 4, 08028 Barcelona, Spain

[9]Catalan Institution of Research and Advanced Studies (ICREA), Passeig de Lluís Companys, 23, 08010, Barcelona, Spain

[10]Centre for Social Evolution, Department of Biology, Universitetsparken 15, University of Copenhagen, Copenhagen DK-2100, Denmark

[11]Trace and Environmental DNA Laboratory, Department of Environment and Agriculture, Curtin University, 6102 Perth, Australia.

[12]Norwegian University of Science and Technology, University Museum, 7491 Trondheim, Norway

[a]s.mak@snm.ku.dk, [b]shyam@snm.ku.dk, [c]christiancaroe@gmail.com, [d]gengchunyu@genomics.cn, [e]shanlin.liu@snm.ku.dk, [f]mikkel.sinding@snm.ku.dk, [g]lukas.kuderna@upf.edu, [h]zhangww@genomics.cn, [i]fushujin@genomics.cn, [j]fgvieira@snm.ku.dk, [k]bent@cbs.dtu.dk, [l]thomas@cbs.dtu.dk, [m]tomas.marques@upf.edu, [n]guojie.zhang@bio.ku.dk, [o]jianghui@genomics.cn, [p]tgilbert@snm.ku.dk

*Correspondence: jianghui@genomics.cn, tgilbert@snm.ku.dk

[†]Contributed equally

Abstract

Background: Ancient DNA research has been revolutionised following development of 'Next Generation' Sequencing platforms. Although a number of such platforms have been applied to ancient DNA samples, the Illumina series are the dominant choice today, mainly because of high production capacities and short read production. Recently a potentially attractive alternative platform for palaeogenomic data generation has been developed, the BGISEQ-500, whose sequence output are comparable with the Illumina series. In this study, we modified the standard BGISEQ-500 library preparation specifically for use on degraded DNA, then directly compared the sequencing performance and data quality of the BGISEQ-500 to the Illumina HiSeq2500 platform, on DNA extracted from eight historic and ancient dog and wolf samples.

Results: The data generated was largely comparable between sequencing platforms, with no statistically significant difference observed for parameters including level and average sequence length of endogenous nuclear DNA, sequence GC content, double stranded DNA damage rate, and sequence clonality. Small significant differences were found in single strand DNA damage rate (δS, slight lower for the BGISEQ-500) and the background rate of difference from the reference genome (θ**,** slightly higher for BGISEQ-500). This may result from the differences in amplification cycles used to PCR amplify the libraries. A significant difference was also observed in the level of endogenous mitochondrial DNA sequenced, although we believe this is likely an artifact of the very low level of endogenous material in three of the samples.

Conclusions: Although we acknowledge our analyses were limited to animal material, our observations suggest that the BGISEQ-500 holds the potential to represent valid and potentially valuable alternative platform for palaeogenomic data generation, that is worthy of future exploration by those interested in the sequencing and analysis of degraded DNA.

Keywords: ancient DNA, BGISEQ-500, Illumina HiSeq 2500, comparative performance

Background

As with many other disciplines, the advent of so-called 'Next Generation Sequencing' (NGS) platforms has revolutionised ancient DNA (aDNA) research. During the era of Sanger sequencing, the dataset within most studies were restricted to short lengths of mtDNA or nuDNA, and at most, if one used multiplexing techniques, one could aim for mitogenomes[1]. However, thanks to NGS techniques, with the right sample and sufficient funds, today practitioners are able to aim for near-complete ancient nuclear genomes (so-called palaeogenomes), even at the population level. While there are now a range of NGS technologies available to choose from, those favoured by the aDNA field are suited to the characteristically short DNA molecules that dominate aDNA extracts [2,3] - thus long-read technologies such as the PacBio (Pacific Biosciences, CA, USA) and Minion (Oxford Nanopore Technologies, Oxford, UK) are not widely used. A range of technologies have been explored in the aDNA context, including the Roche/454 series[4–6], SOLID-4[7], the now discontinued Helicos[8,9] and the Ion Torrent series[10]). The undisputed workhorses however, are the platforms within Illumina series, principally due to a combination of factors that include cost per bp sequence, acceptable sequencing error rate, as well as simply the number of machines available upon which to sequence. In parallel with the expanded use of such platforms in palaeogenomic studies, has been considerable focus on the development and optimisation of (principally) Illumina-related methods to increase data quality and reduce overall cost. Steps taken have included both tailoring library constructions and amplification methods towards the damaged endogenous aDNA, for example through exploiting blunt end ligations[11], removing steps associated with DNA loss (Carøe *et al.*, in review), enzyme choice[12], or even focusing on direct ligation to single stranded DNA[13], as well as improvement in the informatic tools that are used to process the Fastq data generated[14–17].

Today, therefore, Illumina-based sequencing has formed the basis of the overwhelming majority of palaeogenomic studies, including (but not limited to) draft genomes of

humans[18] and related hominids[19–22], animals[9,23–26], plants[27–29] and even pathogens[30–38], population genomic datasets[32,39–44], metagenomic studies[45–48] and even insights into ancient transcriptomes[36,49,50] and epigenomes[51–55]. For recent reviews see [56,57].

Despite this remarkable progress, palaeogenomics still faces one significant limitation - the overall data generation cost. The per base cost of Illumina-based NGS sequencing is falling thanks to improvements relating to flow-cell cluster density, and the generation of longer reads (although for most aDNA this latter point is rarely beneficial). As such, today a modern human 3GB genome can be sequenced to 30x coverage for as little as USD1000[58]. Palaeogenomicists, however, are not so fortunate, given that much (if not in many cases, the majority) of the DNA in most ancient samples is derived from exogenous contaminants[4] such as microbes. While some methodological improvements such as optimised choice of tissue sources[59,60], extraction methods[61–65], and various forms of enrichment help improve the endogenous DNA content[13,54,66–72], costs can still be many fold that for modern DNA data. Thus while attractive to many, the application of palaeogenomics has been largely restricted to the most well-funded research teams and spectacular research questions.

While Illumina has dominated the palaeogenomic sequencing market, in 2016, a new platform emerged that may offer considerable potential to the field - the combinatorial probe-anchor synthesis (cPAS) based BGISEQ-500. The underlying technology combines DNA nanoball (DNB) nanoarrays[73] with polymerase based stepwise sequencing, and its use has recently been exhibited for sequencing small non-coding RNAs[74]. The BGISEQ-500 has several features that suggest it will be it attractive to aDNA users. Firstly, its sequencing read-length capacity (currently up to either Single Read (SR) or Paired End (PE) 100bp) falls within lengths that are acceptable to most palaeogenomicists. Secondly its high throughput - a single 2 channel flow cell can produce at least 500 million single-end reads per channel

(thus up to at least 2 billion PE reads per flow cell) in only a few days. Thirdly, at least the initial stages of the library construction method underlying the BGISEQ-500 are sufficiently close to the methods currently used for Illumina palaeogenomic sequencing, and thus can be easily modified based upon some of the above mentioned previous aDNA-related developments if needed. To fully explore this platform's potential for aDNA, we therefore undertook a direct performance comparison against Illumina technology, by building libraries and sequencing 8 historic and ancient DNA extracts. To both keep the underlying variables as similar as possible, and to exploit a recent (Illumina based) methodological development that (i) simplifies library construction and minimises hands on time and economic cost (Carøe *et al.*, in review) and (ii) performs at least as well as the Meyer and Kircher[11] blunt end method that many palaeogenomicists favour, we did not use the original BGISEQ-500 library method, but rather developed a new protocol based on our recently developed Blunt End Single Tube (BEST) method (Carøe *et al.,* in review). We subsequently undertook a range of bioinformatic analyses aimed at exploring whether the resulting sequence datasets (i.e. Illumina versus BGISEQ-500) exhibited significant differences with regards to a number of parameters that are currently believed important for aDNA studies.

Data Description

DNA was extracted from 8 historic and ancient dog and wolf samples, chosen so as to represent a range of materials that are currently interesting to the palaeogenomics community (Table 1) - in particular with regards to the fragment sizes of the surviving DNA and the range of endogenous DNA content within them. Two of the samples are preserved hides of wolves (*Canis lupus*) of between 91-148 years old, which are believed to contain relatively pure (free of enzymatic inhibitors), although heavily fragmented, DNA (a presumed side effect of the tanning process). The remaining samples are naturally preserved wolf and dog (*Canis familiaris*) remains dated between 600 and 15,000 years old.

Table 1: Samples from which aDNA was extracted

Sample	Original ID	Material	Species	Locality	Age	Extraction
214	CN 214	Hide	Wolf	Uummannaq, Greenland	Before 1869 AD	A
1921	CN 1921	Hide	Wolf	Rosenvinge Bugt, Greenland	1925 AD	A
P84	MGUH VP 3332	Humerus	Wolf	Vølvedal, Greenland	ca. 7,620 YBP	B
P83	NKA 1950x2906	Canine tooth	Dog	GUS, Greenland	ca. 600-1,000 YBP	B
P79	ZMK 350/1982	Tibia	Dog	Qajâ, Greenland	ca. 3,6-2,700 YBP	B
FRC	FRC	Cartilage	Wolf	Tumat, Siberia	ca. 14,164 YBP	C
L	L	Liver	Wolf	Tumat, Siberia	ca. 14,164 YBP	C
M1	M1	Muscle	Wolf	Tumat, Siberia	ca. 14,164 YBP	C

CN 214 was acquired by and registered in the collections of the Natural History Museum of Denmark (NHMD) in 1869. According to museum records, the specimen was shot in Uummannaq, West Greenland prior to 1869. CN 1921 is a wolf that was shot in Rosenvinge Bugt, East Greenland, in 1925, and then subsequently placed in the NHMD collections. MGUH VP 3332 is a bone sample found on the surface 2 m above sea level in 1979 in Vølvedal Peary Land, North Greenland. The specimen has been directly dated to 6,785 ± 100 ^{14}C years (Ua-1346, calibrated age as 7,620 years ago)[75]. NKA 1950x2906 is a tooth sample excavated at the Greenlandic Norse GUS site (Gården Under Sandet / The Farm Beneath the Sand). The site was settled by the Greenlandic Norse and inhabited between ca. 1,000 to 600 YBP[76]. ZMK 350/1982 was excavated from the Saqqaq cultural Paleo-Eskimo site Qajâ. Although the site in general has been dated to between 3,600 and 2,700 YBP, this particular sample is from the earliest occupation layers[77–79]. Lastly samples

FRC, L, and M1 are tissue samples from an extremely well preserved mummified wolf found in the permafrost near the village Tumat in the Sakha Republic, Siberia, Russia. The specimen has been directly dated to 14,223 ± 34 ^{14}C years (ETH-73412, calibrated age as 14,164 years ago).

Following DNA extraction, two aliquots of each extract were constructed into Illumina and BGISEQ-500 libraries, respectively, using identical amounts of starting material (16.3 μl, ~5-50 ng DNA input sample dependent), and then subsequently sequenced to enable bioinformatic comparisons on the data.

Analyses and Discussion

We initially generated between $1.35x10^7$ and $5.94x10^7$ reads per Illumina library, and $2.32x10^7$ $3.39x10^8$ reads per BGISEQ-500 library (Table 2, Supplemental Table S1). The data set supporting the results of this article is available in the ERDA repository (http://www.erda.dk/public/archives/YXJjaGl2ZS1zajh4ZTQ=/published-archive.html).

Following normalisation of the data for read length and depth (Table 2), we found no statistically significant difference between the two datasets with regards to the % endogenous nuclear DNA and average length of endogenous DNA, several of the most important parameters for palaeogenomicists, given their fundamental role in affecting the overall financial cost of a study (Table 3). In contrast, there was a statistically significant difference in the % reads mapping to the mitochondrial genome, with fewer reads mapping in the BGISEQ-500 libraries. However, closer inspection of the data indicates that the total number of mtDNA reads are extremely low for 3 of the samples (Supplemental Table S1), and that for the remainder the numbers are extremely similar. As such, we do not believe there to be much significance behind this observation.

Table 2: Summary data generated

Sample	Platform	Total reads	Normalised % reads retained after adapter removal	Normalized clonality	Normalized endogenous DNA (%)	Normalised length of uniquely mapped reads	θ	δD	δS	GC Content (%)	mtDNA (%)
1921	Illumina	3.08E+07	94.69	0.11	58.73	40.77	0.008	0.008	0.154	51.58	4.51E-03
	BGISEQ-500	5.32E+07	83.97	0.15	59.37	42.14	0.009	0.008	0.132	50.42	2.57E-03
214	Illumina	1.35E+07	99.13	0.07	74.25	49.37	0.008	0.011	0.084	48.60	4.15E-03
	BGISEQ-500	1.98E+08	99.55	0.07	75.51	53.08	0.009	0.012	0.061	47.75	3.11E-04
FRC	Illumina	1.64E+07	99.54	0.03	11.58	73.05	0.008	0.012	0.399	44.01	4.55E-03
	BGISEQ-500	3.39E+08	99.79	0.02	10.22	75.63	0.012	0.012	0.325	43.64	1.98E-04
L	Illumina	2.91E+07	99.63	0.09	1.03	64.65	0.013	0.010	0.415	43.24	6.04E-03
	BGISEQ-500	2.44E+08	99.77	0.08	0.85	66.72	0.013	0.009	0.262	45.99	7.09E-04
M1	Illumina	5.10E+07	99.38	0.06	64.09	72.95	0.007	0.010	0.395	44.27	8.02E-03
	BGISEQ-500	1.79E+08	99.74	0.06	54.80	76.76	0.012	0.010	0.258	43.23	2.31E-03
P79	Illumina	4.18E+07	98.48	0.38	0.07	52.45	0.030	0.012	0.880	43.36	4.65E-06
	BGISEQ-500	8.55E+07	98.08	0.10	0.06	45.77	0.039	0.011	0.550	44.21	6.40E-07
P83	Illumina	2.77E+07	84.67	0.58	0.64	65.78	0.014	0.040	0.842	42.32	4.85E-04
	BGISEQ-500	2.32E+07	86.84	0.32	0.47	66.55	0.017	0.040	0.773	44.30	3.87E-04
P84	Illumina	5.94E+07	98.70	0.31	0.12	54.79	0.015	0.030	0.355	44.42	2.71E-06
	BGISEQ-500	1.57E+08	92.45	0.08	0.10	51.13	0.022	0.020	0.154	47.99	5.15E-07

Table 3: Results of statistical analyses on the data

**Test**	**Paired t-test t**	**P-value**
% Reads retained	-1.131308	0.295
Clonality levels	-1.942886	0.093
% Endogenous DNA	-0.956158	0.371
Endogenous DNA average read length	0.0375544	0.718
θ	3.366145	0.012*
δD	-1.09765	0.309
δS	-3.425669	0.011*
% GC	1.091076	0.311
% mtDNA	-3.073585	0.018*

* Significant at P<0.05

With regards to sequence accuracy, although double strand (δD) sequence damage rates as estimated using MapDamage2.0[15] showed no statistically significant difference, a small, yet statistically significant difference was observed for δS, the single strand damage parameter (lower rate for BGISEQ-500, Table 3). Furthermore, we also observed a small, yet significant difference in the background rate of differences from the reference genome (MapDamage2.0 θ), with slightly higher values observed in the BGISEQ-500 platform (Table 3). We hypothesise that both differences may be explained by the fact that, while the initial steps of the library build methodologies are similar, a greater number of PCR cycles was used to amplify the Illumina libraries. This had a clear effect on overall library complexity, as while there was no statistically significant difference with regards to library clonality levels or the % reads retained after initial filtering (Table 2, Table 3), when we used *preseq*[80] to extrapolate on the library complexity, we observed that in all but one case, the BGISEQ-500 platform provided richer libraries (Figure 1). An alternative explanation could be that because our genome coverage for each sample is relatively low, it is possible that differences in the regions of the genomes sequenced could be driving the differences. Ultimately however, we

feel that full resolution of the differences will require the generation of extensive extra data, and thus more will be learnt in future studies that use the BGISEQ-500.

Figure 1: Library complexity estimated as the number of unique reads as a function of the total number of reads sequenced. These numbers are estimated and extrapolated using the program *preseq*[80]. The total number of reads sequenced for each library can be found in Table 2 and Supplemental Table S1. The solid lines are the estimates for the libraries sequenced on the Illumina HiSeq 2500 platform, while the dotted lines are the estimates for the libraries sequenced on the BGISEQ-500. Each of the 8 samples is represented by a different colour.

We subsequently explored two further parameters that relate to whether there are method specific biases with regards to which part of the genome is sequenced, k-mer frequency and GC content. The k-mer content was largely consistent between methods, with the same samples clustering together. The only exceptions were with samples P83 and 1921, where both methods yielded slightly different k-mer distributions (Figure 2). However, sample P83 shows a very similar k-mer content between libraries and to sample M1, making accurate clustering more challenging. The differences for sample 1921 are more difficult to explain - we note this is the sole BGISEQ-500 library to exhibit lower complexity than its Illumina pair, although it is not clear if/how this may affect the results.

Figure 2 - Heatmap of k-mer counts across libraries. Libraries (columns) were hierarchically clustered based on Pearson correlation. Proportion of each of the 4096 6-mer (rows) are depicted using colours.

GC content was also largely consistent between methods. At a global level, we found no statistically significant difference in the average GC content (Table 2, Table 3), and in more

refined analyses, we observed that the fragment count for the same windows to be well correlated between BGISEQ-500 and Illumina derived reads, both of which are correlated with GC-content (Figure 3, Figure 4). When we calculated the coefficients of determination for the longest scaffold in the assembly (scaffold_0) to minimize potential reference biases on highly fragmented regions, more variable correlations were observed (Figure 4, Table 4). While good correlations were seen for samples 1921, 214, FRC and M1, much lower correlations were observed for samples L, P79, P83 and P84. We believe these differences are most likely attributable to the overall endogenous DNA quality in the samples rather than the platforms' technical performance, as the four worst performing samples have relatively few reads mapping to scaffold_0. Similar results also likely explain why the correlations observed when mapping to the whole genome are even lower still (Table 4).

Table 4: Overview of r^2 values for normalized fragment counts between Illumina and BGISEQ-500 for windows of 100Kb

Sample	r^2 NFC scaffold_0	r^2 NFC whole genome
CN 1921	0.8232490	1.674298e-01
CN 214	0.9268536	6.007588e-01
FRC	0.5558353	5.319692e-01
L	0.1705492	9.810366e-02
M1	0.9178593	9.533171e-01
P79	0.1796964	1.805367e-05
P83	0.0138598	2.648055e-04
P84	0.1497025	5.834542e-03

Figure 3 - Top: Median normalised fragment count (NFC) per 100Kb windows with 10Kb offset for the sample 214 along scaffold_0. The solid line shows Illumina data,

the dotted line shows BGISEQ-500 data. Bottom: Percentage GC calculated over the same the same windows as in the upper panel.

Figure 4: Median normalised fragment count (NFC) of Illumina vs. BGISEQ-500 for all samples in windows of 100Kb with an offset of 10Kb along scaffold_0. The color of each point corresponds to the windows' GC content. For the high quality samples (1921, 214, FRC, M1) a very good correlation of NFC between the two platforms can be observed. Fragment count seems to be correlated with GC-content.

Our final analysis explored CNV levels, although as mentioned above, the low genomic coverage of the data makes CNV analyses challenging. Nevertheless, the r^2 values for the comparisons that pass our quality control range from 0.35-0.96 (Table 5). Furthermore, the observation of particular DNA extractions with excellent concordance values despite the nature of our experiment, make it tempting to speculate that indeed both technologies are viable for high quality CN calls. For example, using 36-mers and accounting for all possible placements of a 36-mer, the sample M1 has a coverage of above 1x on both platforms. Ultimately however, it is not possible to discern from the present data whether the observed variation in CN calls in the samples is due to differences in the sequencing platforms or in the nature of the libraries, thus these results should be taken as preliminary, pending future validation.

Table 5: Coefficients of determination for copy number in the same genomic windows between platforms, for all extracts at varying resolution.

	CW Size					
**Sample**	1000Kbp	100Kbp	50Kbp	10Kbp	5Kbp	1Kbp
214	0.905[a]	0.331[a]	0.354[a]	0.506[a]	0.519[b]	0.433[c]
1921	0.963[a]	0.384[a]	0.392[a]	0.428[a]	0.432[b]	0.393[c]
FRC	0.582[a]	0.847[a]	0.870[a]	0.873[b]	0.870[c]	0.783[c]
L	0.941[b]	0.957[c]	0.964[c]	0.958[c]	0.955[c]	ND
M1	0.665[a]	0.943[a]	0.952[a]	0.953[a]	0.950[a]	0.910[b]
P79	0.672[b]	ND	ND	ND	ND	ND
P83	0.203[b]	0.003[c]	0.004[c]	0.003[c]	0.002[c]	ND
P84	0.919[b]	0.001[c]	0.001[c]	ND	ND	ND

[a]Denotes a pass of quality control (visual inspection of read depth density in control regions and proper SW/CW and LW/CW ratios. [b]Denotes suboptimal quality, e.g. not perfectly symmetrical, bell shaped read depth distribution in control regions. ND=insufficient Data for at least one platform. [c]Denotes failed QC for at least one platform.

Potential Implications

Our study represents the first exploration of the applicability of the BGISEQ-500 as an alternative sequencing platform to the Illumina series for palaeogenomic sequencing, and in doing so we present a library build protocol to generate such data. Although our study is based around only 8 specimens, given their ranges of endogenous DNA content (<1-75%) and normalised average endogenous DNA sequence lengths (ca 42-76 bp) are typical of many other ancient samples, we anticipate that our results be indicative of the platform on such material general. Overall the results are extremely promising - the BGISEQ-500's performance is comparable over all parameters tested, with the exception of the very slightly

elevated error rate observed (although in contrast we observe higher library complexity and lower δS, thus overall feel this will not represent a major concern to palaeogenomic studies). As such, we anticipate that our findings will stimulate considerable interest in its use by palaeogenomic research teams attempting to reconstruct ancient genomes and transcriptomes, and look forward to future exploration of its potential across a wider range of ancient substrates.

Methods

DNA extraction

DNA was extracted using one of three different methods (designated A, B, C, Table 1), as deemed appropriate for the choice of tissue. Methods A and C involved digestion in a proteinase K containing buffer following[59], while method B involved digestion in a proteinase K-urea buffer following[81]. All samples were pre-digested at 56 °C for 1 hour, after which the buffer was changed and then a second 12 hour digest was performed. Digests from method A used organic solvents (phenol:chloroform) and Qiagen MinElute columns (Qiagen, Hilden, DE), following Carøe *et al.* (Carøe *et al.,* in review). Digests from methods B and C were centrifuged at 6000 xG for 1 minute, after which 500 µl supernatant was mixed 1:8 with a binding buffer as detailed in Allentoft *et al*.[40], then centrifuged through Monarch DNA Cleanup Columns (New England Biolabs, Massachusetts, USA). DNA bound to the columns was washed with 800 µl buffer PE (Qiagen), then eluted using two washes in 17 µl buffer EB (Qiagen) - each with an incubation for 5 minutes at 37 °C. Prior to library construction small aliquots of each extract were analysed on an Agilent 2200 TapeStation HS chip (Agilent Technologies, Palo Alto, California, USA) for fragment size estimation and molar concentration.

Library construction

Two aliquots of each extract were constructed into Illumina and BGISEQ-500 libraries, respectively, using identical amounts of starting material (16.3 µl, ~5-50 ng DNA input

sample dependent) (Supplemental Table S2). Library blanks and index PCR blanks were also included to evaluate the potential contaminations during the library building process. Illumina libraries were constructed using a method based upon the recently published single tube 'BEST' protocol, largely following Carøe *et al.* (Carøe *et al.,* in review) although with some modifications (Supplemental File F1). To both enable direct comparison of the sequencing methods, we chose not to use the conventional BGISEQ-500 library construction protocol. Rather, given the similarities between the initial processes of library construction between both methods (DNA end repair and adapter ligation), we modified the BEST protocol to be BGISEQ-500 compatible. Specifically, the standard Illumina compatible adapters were replaced with BGISEQ-500 compatible adapters AD1 and AD2 (Supplemental Table S3). These adapters were synthesised as two pairs of complementary oligonucleotides (AD1_Long and AD1_Short, and AD2_Long and AD2_Short, respectively), then prepared into the final adapters, AD1 and AD2. Specifically, adapters were first diluted to 500μM with 1X TE buffer (10mM Tris-HCl, 1mM EDTA, pH 8.0, Sigma-Aldrich). Subsequently, an equimolar concentration of each pair of Long and Short adapters was mixed together and hybridized through incubation at 95 °C for 1 min, followed by a decrease in temperature with 0.1 °C/s from 95 °C to 12 °C. After hybridization, adapters AD1 and AD2 were mixed and diluted at a concentration of 10 μM prior to their use in the library construction. We additionally designed BGISEQ-500 compatible library amplification primers for use in the library amplification steps, that included 8 alternate sequencing indices in the reverse primers (Supplemental Table S3).

Following the final Bst fill-in step during library build, all libraries were mixed with 1:5 volume of PB binding buffer (Qiagen) and purified using Monarch® DNA clean up columns,then washed with 750 μl buffer PE (Qiagen) and eluted in 40 μl buffer EB (Qiagen) after a 5 minute incubation at 37 °C.

Illumina library PCR amplification and sequencing

Quantitative real-time PCR (qPCR) was used to estimate the required number of cycles during library index PCR. Each qPCR was performed in a 20 µl reaction volume using 1:20 dilution of purified library template, 0.2 mM dNTPs (Invitrogen), 0.04 U/µl AmpliTaq Gold DNA polymerase (Applied Biosystems, Foster City, California, USA), 2.5 mM $MgCl_2$ (Applied Biosystems), 1X GeneAmp® 10X PCR Buffer II (Applied Biosystems), 1 µl SYBR Green (Invitrogen, Carlsbad, California, USA), 0.2 µM forward and reverse primers mixture (IS7 and IS8 primers [11]) and 13.48 µl AccuGene molecular biology water (Lonza). qPCR cycling conditions were 95 °C for 10 min; following by 40 cycles of 95 °C for 30 s; 60 °C for 60 s, and 72 °C for 60 s using the MX3005 qPCR machine (Agilent).

Post qPCR, library index amplifications were performed in 100 µl PCR reactions that contained 20 µl of purified library, 0.2 mM dNTPs (Invitrogen), 0.1 U/µl AmpliTaq Gold DNA polymerase (Applied Biosystems), 2.5 mM $MgCl_2$ (Applied Biosystems), 1X GeneAmp® 10X PCR Buffer II (Applied Biosystems), 0.4 mg/ml BSA (New England Biolabs Inc), 0.2 µM of each forward (Illumina InPE 1.0 forward) and custom made reverse primers, and 51.2 µl AccuGene molecular biology water (Lonza, Basel, CH). PCR cycling conditions were: initial denaturation at 95 °C for 12 min followed by 13-21 cycles of 95 °C for 30 s, 60 °C for 30 s and 72 °C for 40 s, and a final elongation step at 72 °C for 5 min. Post-PCR, libraries were purified with QiaQuick columns (Qiagen) and eluted with 30 µl buffer EB (Qiagen) after an incubation for 10 min at 37 °C. Small aliquots of this purified product were used for quantification and fragment size estimation using the High-Sensitivity DNA Assay for the Bioanalyzer 2100 (Agilent). Subsequently, a final purification using the AMpure XP system (Agentcourt, Beckman Counter, Indianapolis, USA) with 1.8X beads:library ratio, in order to remove any persisting primer dimers or other molecules with a fragment size of <100 bp. Lastly, libraries were pooled in equimolar concentrations (~9.4 nM) and sequenced on the

Illumina HiSeq platform in 80 bp single read mode by The Danish National High-Throughput DNA Sequencing Centre.

BGISEQ-500 library PCR amplification

Initial processing steps for the purified BGISEQ-500 libraries were largely similar to that used on the Illumina libraries, although with the following modifications. Firstly the libraries were qPCR quantified using the *CommonprimerBGI forward* primer and one of the indexed reverse primers (Supplemental Table S3). Secondly, subsequent index PCR amplifications used 8-15 cycles, with *CommonprimerBGI forward* primer and the indexed reverse primers (Supplemental Table S3). Thirdly, because several of the BGISEQ-500 libraries exhibited residual adapter dimers after the initial purification post index PCR, each purified BGISEQ-500 library was split to 2 aliquots (~12.5 µl each), and one of each aliquot was subject to an extra purification to remove any residual primer dimers (Supplemental Table S2). Each of these aliquots was sequenced independently. We note that several of the extra purified libraries showed small improvements with regards to overall adapter dimer content in the generated sequence (Supplemental Table S4), and our initial impression is that this extra purification step may be worth undertaking if high levels of adapter dimers are found post index PCR.

BGISEQ-500 library circularisation and sequencing

All amplified libraries were subsequently sent to BGI for circularisation and sequencing on the BGISEQ-500 platform. For circularisation, PCR products with different barcodes were pooled together at equimolar concentration to yield a final amount of 80 ng. Pools contained both the samples relevant to this study as well as those from other projects (Supplemental Table S5). Each pool was subsequently heat denatured and the single strand DNA were mixed with MGIEasyTM DNA Library Prep Kit V1 (PN:85-05533-00, BGI, Shenzhen, China), containing 5 µl splint oligo, 6 µl splint Buffer, 0.6 µl ligation Enhancer, 0.2 µl ligation Enzyme and NF water) to form a 60 µl reaction system, which was subsequently incubated at 37 °C

for 30 min. Lastly, 20 µl of each single-circle-library pool was used as input to prepare the DNA Nano Ball (DNB). Each pool was then sequenced on 1 lane, using 100SR chemistry with BGISEQ-500RS High-throughput sequencing kit (PN: 85-05238-01, BGI). Post sequencing, the data was automatically demultiplexed by index.

Data analyses

The raw reads obtained from the HiSeq 2500 and BGISEQ-500 were analysed using FastQC[82] to compute the quality metrics of the reads, such as, base sequence qualities, base sequence content, %GC, and sequence composition. With the exception of the analysis on the standard versus extra-purified BGISEQ-500 libraries (Supplemental Table S4), both BGISEQ-500 libraries from each extract were treated as a single dataset. We also compared the quality metrics of the reads from the same samples across the two platforms to ensure that the sequencing platform did not have a large impact on the quality metrics of the reads.

Once the read qualities were verified using FastQC, we used the PALEOMIX pipeline[16] to trim the adapter sequences, trim Ns and low quality bases from the ends of reads, estimate ancient DNA damage, and finally map the trimmed reads to the reference genome. The individual steps of the pipeline are detailed below. We highlight that the values presented in Table 2 are normalised to account for sequencing read depth and length, while Supplemental Table S1 contains both the original, and normalised values.

Adapter removal and trimming

The first step of the initial processing of the reads involved trimming the adapter sequences from the ends of the reads. Since the samples consist of degraded DNA, many of the sequenced reads contain the platform specific adapters at the 3' end of the reads. AdapterRemoval (v2.1.3)[83] was used to trim the adapter sequences from the ends of the reads using the default mismatch rate of 1/3. In addition, bases with a quality score less than

2 and unidentified bases (Ns) at the ends of reads were trimmed. Finally, only reads that were longer than 25 bases were retained for downstream analyses.

Mapping, indel realignment and duplicate removal

The trimmed reads were mapped to the wolf reference genome (Gopalakrishnan *et al.*, in review, available upon request) using the mem algorithm in bwa (v0.7.10), using the default settings for the mapping algorithm. The mapped reads were subsequently processed using the GATK (v3.3.0) indel realigner[84,85], to fix the alignment issues arising from the presence of short indels at the beginnings and ends of reads. Since there are no catalogs of indel variations in the species included in this study, the realignment step was done using a set of indels within each sample. After the indel realignment step, the PCR duplicates were removed from the alignments using the MarkDuplicates program from Picard tools (v1.128)[86].

DNA Damage patterns

The DNA damage patterns and parameters were estimated using mapDamage (v2.0.6)[15] using a subsample of 100,000 reads from the set of mapped reads. The three main parameters estimated using mapDamage were θ, δD, and δS. δD and δS estimate the probability of cytosine deamination (driven by hydrolytic DNA damage) in a double (δD) and single (δS) stranded context, while θ estimates the background rate of difference between the reference and sample after accounting for DNA damage. Using these estimated parameters, the base qualities of putatively damaged bases were recalibrated to a lower score. The program was also used to compute the relative abundance of C→T changes at the 3' ends and A→G changes at the 5' ends of the reads and compare them across the two platforms.

Clonality, endogenous DNA content and library complexity estimation

The clonality of each library was computed from the reads that were identified by the MarkDuplicates program during the duplicate identification and removal step. The clonality was computed as the ratio of the number of reads retained after duplicate removal and the number of reads retained after the adapter removal and trimming step. The endogenous content of the library was computed as the ratio of the number of reads mapping uniquely to the reference genome and the number of reads retained after adapter removal. Note that this is one possible definition of the endogenous content, here defined as the proportion of usable reads obtained from a library, and the numbers given in Table 2 and Supplemental Table S1 will allow you to compute the values for other definitions of endogenous content.

The complexity of each library was estimated, and extrapolated, using the library complexity extrapolation model in the program *preseq*[80], which uses a non-parametric Bayesian Poisson model to estimate the gain in number of unique fragments when the library is sequenced deeper. Instead of using the aligned reads to estimate the library complexity, we used the counts of the number of duplicates in the bams generated by paleomix as input to *preseq*. The library complexity was estimated up to a maximum of a total of 10 billion reads sequenced per library.

Mapping to the wolf mitochondrial genome

Since the draft de novo wolf genome does not contain information on scaffolds that are annotated as belonging to the mitochondria, we could not identify reads that mapped to the mitochondrial genome using the initial set of mapped reads. To overcome this problem, we downloaded a complete mitochondrial genome from NCBI (GenBank Accession: AM711902, [87]) and mapped the adapter trimmed reads to this complete mitochondrial genome. The same steps, including indel realignment and DNA damage related recalibration of quality scores was performed for the reads aligned to the mitochondria.

K-mer frequency

To compare the sequence content of the reads obtained from the two sequencing platforms, we computed the k-mer frequencies in the reads from the same sample using the two technologies. Since the raw reads are enriched in adapter sequences and do not accurately reflect the sequence content of the underlying endogenous DNA molecules in the library, we restricted the k-mer analysis to reads that mapped to the genome after going through both adapter trimming and duplicate read removal. For each library, we sampled 100000 reads from the reads mapped to the reference genome using samtools (v1.2)[88,89] and seqtk (v1.0)[90]. From these subsampled reads, we computed the 6-mer frequencies using jellyfish[91].

Relative abundance vs GC content

The relationship between read abundance in a given genomic region and its GC content, is well known and characterized for the Illumina platform[92]. For methods that depend upon depth of coverage or fragment count, such as measuring absolute copy number or expression levels, this bias needs to be taken into consideration and corrected for, otherwise, its magnitude might confound the signal in question. We therefore compared the GC content of the mapped endogenous DNA for the two platforms in several ways. Firstly, the basic GC percentage was calculated from all endogenous reads. Secondly, we partitioned the reference genome into bins of 100 Kbps, with an offset of 10 Kbps, and calculated the GC percentage of each bin. We then mapped all datasets onto the reference, and counted the number of mapped fragments in each bin. To account for differences in sequencing depth, we normalized the number of mappings by the median number of mappings for each extract. Lastly, we also calculated coefficients of determination for the longest scaffold in the assembly (scaffold_0), to minimize potential reference biases on highly fragmented regions.

CNV on low coverage data

Fluctuations in depth of sequencing coverage can be used to generate personal genome wide copy number (CN) maps of an individual, as read depth is known to strongly correlate with copy number for several platforms[93]. We sought to assess whether the same techniques might be applied to data generated on the BGISEQ-500. To this end, we generated individual genome wide CN maps of all extracts and both platforms in varying window sizes from 1Kbp to 1Mbp to account for fluctuation in coverage, and checked concordance between them. It is worth noting, that using ancient DNA libraries poses a particular challenge to this assessment, as some inherent characteristics of this type of data (such as unequal degradation, fragmentation or clonality during library preparation) make it difficult to pinpoint the source of variability between two call sets for a given extract, given a lack of concordance. Specifically, low effective coverage and poor DNA quality make high-resolution maps not feasible for many of the libraries used in this part of the project.

We masked out any repeats in the reference assembly, as identified by both *repeat masker*[94] and tandem *repeat finder*[95]. Additionally, to identify repeats that have been potentially missed by the aforementioned algorithms, we chopped up the masked assembly into 36-mers with an offset of 5bp. These were then mapped back onto the assembly using GEM[96] with a maximum divergence set to 95% and retaining all possible mappings. All 36-mers with more than 20 placements along the genome were additionally masked out. We then generated non-overlapping 36-mers from the production reads, and mapped them onto the extensively masked reference assembly using GEM, allowing for a maximum divergence of 95% and retaining all possible placements. To call absolute copy number, the reference was portioned in windows of 1, 5, 10, 50, 100, 1000 Kbps of non-overlapping, non-repetitive sequences with *mrCanavar*[93], meaning that the genomic coordinates of the windows may span more than the window size if repeats are present within it. Importantly, as reads may not properly map at the boundaries of maskings, we introduced an additional padding of 36 bp. We then iteratively excluded all windows that represent outliers with respect to a normal distribution, to identify a set of ‘control regions’. After correcting for GC content, the median

depth of coverage in these control regions was used to normalize all windows and thus assign an absolute copy number to them. The concordance was calculated as the coefficient of determination of a linear model over corresponding to windows of the same extract between the two platforms. Additional quality control involved visually inspecting the normalized read depth distribution of the aforementioned control regions. In a good sample, this should be a symmetrical, bell-shaped curve centered at 2. We visually inspected all distributions and classified them as good, neutral or bad, based on shape and symmetry. In addition to the aforementioned windows (called Copy-Windows, CW), we also calculated normalized read depths in windows the same size of CW in terms of non-repetitive sequence, with a fixed offset of the window size, but including repetitive sequence, (called Short-Windows, SW), and windows 5 times the size of the Copy Window (called Long-Windows, LW), with an offset of 5 times the size of a copy window, but including repetitive sequence. As an additional quality control, the ratios of read depth of SW/CW should be around 1, and the ratio of read depths of LW/CW around 5, given proper sampling of the genome.

Availability of Supporting Data

The data set supporting the results of this article is available in the ERDA repository, http://www.erda.dk/public/archives/YXJjaGl2ZS1zajh4ZTQ=/published-archive.html

Additional Files

Supplemental File F1 – Improvements to original BEST library building protocol

(see additional file)

Supplemental Table S1 - Full sequence data information

(see additional file)

Supplemental Table S2 - Sequence library identifiers

DNA extract	Illumina Library ID	BGISEQ-500 Library ID (standard)	BGISEQ-500 Library ID (extra purification)
214	214	z_214	z_214p
1921	1921	z_1921	z_1921p
P79	P79	z_P79	z_79p
P83	P83	z_P83	z_P83p
P84	P84	z_P84	z_P84p
FRC	FRC	z_FRC	z_FRCp
L	L	z_L	z_Lp
M1	M1	z_M1	z_M1p

Supplemental Table S3 - The sequences of BGISEQ-500 adapters and index primers used in this study.

Name	Sequence (5' -> 3')	Modification
BGISEQ-500 Adapters		
AD1_Long	TTGTCTTCCTAAGACCGCTTGGCCTCCGACTT	
AD1_Short	AAGTCGGAGGCC	
AD2_Long	TTGTCTTCCTAAGGAACGACATGGCTACGATCCGACTT	
AD2_Short	AAGTCGGATCGT	
*Index Primers**		
IndexprimerBGI_1	TGTGAGCCAAGGAGTTG**ACAGTATTTA**TTGTCTTCCTAAGACCGC	
IndexprimerBGI_2	TGTGAGCCAAGGAGTTG**AATTAATTCC**TTGTCTTCCTAAGACCGC	
IndexprimerBGI_3	TGTGAGCCAAGGAGTTG**CTGAGTGACT**TTGTCTTCCTAAGACCGC	
IndexprimerBGI_4	TGTGAGCCAAGGAGTTG**ATTCCGTCAG**TTGTCTTCCTAAGACCGC	
IndexprimerBGI_5	TGTGAGCCAAGGAGTTG**AACTATCTAA**TTGTCTTCCTAAGACCGC	
IndexprimerBGI_6	TGTGAGCCAAGGAGTTG**GGAAGGACCA**TTGTCTTCCTAAGACCGC	
IndexprimerBGI_7	TGTGAGCCAAGGAGTTG**TTATAGAGAG**TTGTCTTCCTAAGACCGC	
IndexprimerBGI_8	TGTGAGCCAAGGAGTTG**GTACAAAGGG**TTGTCTTCCTAAGACCGC	
Commonprimer BGI forward	GAACGACATGGCTACGA	5’ Phosphate

***Variable 10bp indices indicated in bold.**

Supplemental Table S4 - Adapter dimer content of initial, and extra purified BGISEQ-500 libraries

Sample	Standard library	Extra purified
1921	82.38%	88.66%
214	99.51%	99.58%
P79	97.47%	98.69%
P83	71.96%	90.90%
P84	87.74%	98.00%
FRC	99.83%	99.72%
L	99.52%	99.95%
M1	99.91%	99.69%

Supplemental Table S5 - Library pooling for BGISEQ-500 library circularisation reactions

Lane number	Pool (ssCir)	Library (Index)
1	ancient_1	Lp (1), M1 (2), 214 (5), FRC (7), others (17-24)
2	ancient_2	P84p (1), M1p (2), 214p (5), 1921 (6), others (9-16)
3	ancient_3	L (1), P83p (2), P79 (3), libCH2* (5), 1921p (6), FRCp (7), others (9-16)
4	ancient_4	P84 (1), P83 (2), P79p (3), libCH1* (4), 214 (5), FRC (7), others (9-16)

*libCH1 and libCH2 are control blank libraries that did not yield any data post sequencing.

Abbreviations

aDNA - Ancient DNA; BEST - Blunt End Single Tube; CNV - Copy Number Variation; CN - Copy Number; cPAS - Combinatorial Probe-Anchor Synthesis; CW - Copy Windows; DNB - DNA nanoball; GB - Gigabase; LW - Long Windows; NHMD - Natural History Museum of Denmark; NFC - Normalised Fragment Count; NGS - Next Generation Sequencing; PE - Paired End; SR - Single Read; SW - Short Windows; YBP - Years Before Present; δS - MapDamage 2.0 single strand DNA damage rate; δD- MapDamage 2.0 double strand DNA damage rate; θ - MapDamage 2.0 DNA damage corrected error rate

Competing Interests

The authors declare that Hui Jiang, Chunyu Geng, Guojie Zhang, Wenwei Zhang, Shujin Fu and Shanlin Liu are employees of BGI.

Authors Contributions

M.T.P.G., G.Z. and H.J. conceived the study with critical input from S.L., C.C. and S.S.T.M.. C.C. adapted the BGISEQ-500 library construction method for aDNA. S.S.T.M. prepared the aDNA libraries. M-H.S.S. extracted the aDNA. C.G., W.Z., and S.F. performed BGISEQ-500 library circularisation, ssDNA synthesis and the BGISEQ-500 sequencing. S.G., F.G.V., L.F.K.K. and T.M.B. analysed the data with assistance from S.L., T.S.P. and B.P. M.T.P.G. drafted the manuscript, with input from all authors.

Acknowledgements

The authors would like to acknowledge the assistance of the Danish National High-Throughput Sequencing Centre for assistance in Illumina data generation, and ERC Consolidator Grant (681396 – Extinction Genomics), the Marie Skłodowska-Curie Actions (H2020-MSCA-ETN-643063 ‘Microwine’), Danish Council for Independent Research (4005-00107 Wine-ometrics), China National GeneBank and BGI-Shenzhen China for funding. We also gratefully acknowledge the Danish National Supercomputer for Life Sciences –

Computerome (computerome.dtu.dk) for the computational resources to perform the sequence analyses. L.F.K.K is supported by an FPI fellowship associated to BFU2014-55090-P (FEDER), T.M.B. is supported by MINECO BFU2014-55090-P (FEDER) and BFU2015-6215-ERC, U01 MH106874 grant and Secretaria d’Universitats i Recerca del Departament d’Economia i Coneixement de la Generalitat de Catalunya.

References

1. Krause J, Dear PH, Pollack JL, Slatkin M, Spriggs H, Barnes I, *et al*. Multiplex amplification of the mammoth mitochondrial genome and the evolution of Elephantidae. Nature. Nature Publishing Group; 2006;439:724–7.

2. Lindahl T. Instability and decay of the primary structure of DNA. Nature. Nature Publishing Group; 1993;362:709–15.

3. Pääbo S. Ancient DNA: extraction, characterization, molecular cloning, and enzymatic amplification. Proc. Natl. Acad. Sci. U. S. A. 1989;86:1939–43.

4. Poinar HN, Schwarz C, Qi J, Shapiro B, Macphee RDE, Buigues B, *et al*. Metagenomics to paleogenomics: large-scale sequencing of mammoth DNA. Science. American Association for the Advancement of Science; 2006;311:392–4.

5. Miller W, Drautz DI, Ratan A, Pusey B, Qi J, Lesk AM, *et al*. Sequencing the nuclear genome of the extinct woolly mammoth. Nature. Nature Publishing Group; 2008;456:387–90.

6. Gilbert MTP, Kivisild T, Gronnow B, Andersen PK, Metspalu E, Reidla M, *et al*. Paleo-Eskimo mtDNA Genome Reveals Matrilineal Discontinuity in Greenland. Science. American Association for the Advancement of Science; 2008;320:1787–9.

7. Keller A, Graefen A, Ball M, Matzas M, Boisguerin V, Maixner F, *et al*. New insights into the Tyrolean Iceman's origin and phenotype as inferred by whole-genome sequencing. Nat. Commun. Nature Publishing Group; 2012;3:698.

8. Orlando L, Ginolhac A, Raghavan M, Vilstrup J, Rasmussen M, Magnussen K, *et al*. True single-molecule DNA sequencing of a Pleistocene horse bone. Genome Res. 2011;21:1–51.

9. Orlando L, Ginolhac A, Zhang G, Froese D, Albrechtsen A, Stiller M, *et al*. Recalibrating *Equus* evolution using the genome sequence of an early Middle Pleistocene horse. Nature. Nature Publishing Group; 2013;1–8.

10. Murray DC, Pearson SG, Fullagar R, Chase BM, Houston J, Atchison J, *et al*. High-throughput sequencing of ancient plant and mammal DNA preserved in herbivore middens. Quat. Sci. Rev. Elsevier Ltd; 2012;58:135–45.

11. Meyer M, Kircher M. Illumina sequencing library preparation for highly multiplexed target capture and sequencing. Cold Spring Harb. Protoc. 2010;2010:db.prot5448.

12. Seguin-Orlando A, Hoover CA, Vasiliev SK, Ovodov ND, Shapiro B, Cooper A, *et al*. Amplification of TruSeq ancient DNA libraries with AccuPrime Pfx: consequences on nucleotide misincorporation and methylation patterns. STAR: Science & Technology of Archaeological Research. 2015;1:1–9.

13. Gansauge M-T, Meyer M. Single-stranded DNA library preparation for the sequencing of ancient or damaged DNA. Nat. Protoc. Nature Publishing Group; 2013;8:737–48.

14. Ginolhac A, Rasmussen M, Gilbert MTP, Willerslev E, Orlando L. mapDamage: testing for damage patterns in ancient DNA sequences. Bioinformatics. 2011;27:2153–5.

15. Jónsson H, Ginolhac A, Schubert M, Johnson PLF, Orlando L. mapDamage2.0: fast approximate Bayesian estimates of ancient DNA damage parameters. Bioinformatics. 2013;29:1682–4.

16. Schubert M, Ermini L, Der Sarkissian C, Nsson HAKJO, Ginolhac AEL, Schaefer R, *et al*. Characterization of ancient and modern genomes by SNP detection and phylogenomic and metagenomic analysis using PALEOMIX. Nat. Protoc. Nature Publishing Group; 2014;9:1056–82.

17. Peltzer A, Jäger G, Herbig A, Seitz A, Kniep C, Krause J, *et al*. EAGER: efficient ancient genome reconstruction. Genome Biol. 2016;17:60.

18. Rasmussen M, Li Y, Lindgreen S, Pedersen JS, Albrechtsen A, Moltke I, *et al*. Ancient human genome sequence of an extinct Palaeo-Eskimo. Nature. Nature Publishing Group; 2010;463:757–62.

19. Green RE, Krause J, Briggs AW, Maricic T, Stenzel U, Kircher M, *et al*. A draft sequence of the Neandertal genome. Science. American Association for the Advancement of Science; 2010;328:710–22.

20. Meyer M, Kircher M, Gansauge MT, Li H, Racimo F, Mallick S, *et al*. A High-Coverage Genome Sequence from an Archaic Denisovan Individual. Science [Internet]. American Association for the Advancement of Science; 2012; Available from: http://www.sciencemag.org/cgi/doi/10.1126/science.1224344

21. Prüfer K, Racimo F, Patterson N, Jay F, Sankararaman S, Sawyer S, *et al*. The complete genome sequence of a Neanderthal from the Altai Mountains. Nature. Nature Publishing Group; 2014;505:43–9.

22. Reich D, Green RE, Kircher M, Krause J, Patterson N, Durand EY, *et al*. Genetic history of an archaic hominin group from Denisova Cave in Siberia. Nature. Nature Publishing Group; 2010;468:1053–60.

23. Park SDE, Magee DA, McGettigan PA, Teasdale MD, Edwards CJ, Lohan AJ, *et al*. Genome sequencing of the extinct Eurasian wild aurochs, *Bos primigenius*, illuminates the phylogeography and evolution of cattle. Genome Biol. BioMed Central Ltd; 2015;1–15.

24. Skoglund P, Ersmark E, Palkopoulou E, Dalén L. Ancient Wolf Genome Reveals an Early Divergence of Domestic Dog Ancestors and Admixture into High-Latitude Breeds. Curr. Biol. Elsevier Ltd; 2015;1–6.

25. Frantz LAF, Mullin VE, Pionnier-Capitan M, Lebrasseur O, Ollivier M, Perri A, *et al*. Genomic and archaeological evidence suggest a dual origin of domestic dogs. Science. American Association for the Advancement of Science; 2016;352:1228–31.

26. Palkopoulou E, Mallick S, Skoglund P, Enk J, Rohland N, Li H, *et al*. Complete Genomes Reveal Signatures of Demographic and Genetic Declines in the Woolly Mammoth. Curr. Biol. Elsevier Ltd; 2015;1–7.

27. Ramos-Madrigal J, Smith BD, Víctor Moreno-Mayar J, Gopalakrishnan S, Ross-Ibarra J, Gilbert MTP, *et al*. Genome Sequence of a 5,310-Year-Old Maize Cob Provides Insights into the Early Stages of Maize Domestication. Curr. Biol. [Internet]. Elsevier; 2016 [cited 2016 Nov 19];0. Available from: http://www.cell.com/current-biology/abstract/S0960-9822(16)31120-4?_returnURL=http%3A%2F%2Flinkinghub.elsevier.com%2Fretrieve%2Fpii%2FS0960982216311204%3Fshowall%3Dtrue

28. Mascher M, Schuenemann VJ, Davidovich U, Marom N, Himmelbach A, Bner SHU, *et al*. Genomic analysis of 6,000-year-old cultivated grain illuminates the domestication history of barley. Nat. Genet. Nature Publishing Group; 2016;1–7.

29. Vallebueno-Estrada M, Rodríguez-Arévalo I, Rougon-Cardoso A, Martínez González J, García Cook A, Montiel R, *et al*. The earliest maize from San Marcos Tehuacán is a partial domesticate with genomic evidence of inbreeding. Proc. Natl. Acad. Sci. U. S. A. [Internet]. 2016; Available from: http://dx.doi.org/10.1073/pnas.1609701113

30. Martin MD, Cappellini E, Samaniego JA, Zepeda ML, Campos PF, Seguin-Orlando A, *et al*. Reconstructing genome evolution in historic samples of the Irish potato famine pathogen. Nat. Commun. Nature Publishing Group; 2013;4:2172–2172.

31. Yoshida K, Schuenemann VJ, Cano LM, Pais M, Mishra B, Sharma R, *et al*. The rise and fall of the *Phytophthora infestans* lineage that triggered the Irish potato famine. Elife. 2013;2:e00731–e00731.

32. Martin MD, Vieira FG, Ho SYW, Wales N, Schubert M, Seguin-Orlando A, *et al*. Genomic Characterization of a South American *Phytophthora* Hybrid Mandates Reassessment of the Geographic Origins of *Phytophthora infestans*. Mol. Biol. Evol. SMBE; 2016;33:478–91.

33. Schuenemann VJ, Singh P, Mendum TA, Krause-Kyora B, Jäger G, Bos KI, *et al*. Genome-wide comparison of medieval and modern *Mycobacterium leprae*. Science. American Association for the Advancement of Science; 2013;341:179–83.

34. Bos KI, Schuenemann VJ, Golding GB, Burbano HA, Waglechner N, Coombes BK, *et al*. A draft genome of *Yersinia pestis* from victims of the Black Death. Nature. Nature Publishing Group; 2011;1–5.

35. Rasmussen S, Allentoft ME, Nielsen K, Orlando L, Sikora M, Sjögren K-G, *et al*. Early Divergent Strains of *Yersinia pestis* in Eurasia 5,000 Years Ago. Cell. Elsevier; 2015;1–13.

36. Smith O, Clapham A, Rose P, Liu Y, Wang J, Allaby RG. A complete ancient RNA genome: identification, reconstruction and evolutionary history of archaeological Barley Stripe Mosaic Virus. Sci. Rep. [Internet]. Nature Publishing Group; 2014;4. Available from: http://www.nature.com/doifinder/10.1038/srep04003

37. Wagner DM, Klunk J, Harbeck M, Devault A, Waglechner N, Sahl JW, *et al*. *Yersinia pestis* and the Plague of Justinian 541–543 AD: a genomic analysis. Lancet Infect. Dis. 2014;14:319–26.

38. Maixner F, Krause-Kyora B, Turaev D, Herbig A, Hoopmann MR, Hallows JL, *et al*. The 5300-year-old Helicobacter pylori genome of the Iceman. Science. American Association for the Advancement of Science; 2016;351:162–5.

39. da Fonseca RR, Smith BD, Wales N, Cappellini E, Skoglund P, Fumagalli M, *et al*. The origin and evolution of maize in the Southwestern United States. Nature Plants. Nature Publishing Group; 2015;1:14003.

40. Allentoft ME, Sikora M, Sjögren K-G, Rasmussen S, Rasmussen M, Stenderup J, *et al.* Population genomics of Bronze Age Eurasia. Nature. Nature Publishing Group; 2015;522:167–72.

41. Skoglund P, Malmström H, Raghavan M, Stora J, Hall P, Willerslev E, *et al*. Origins and Genetic Legacy of Neolithic Farmers and Hunter-Gatherers in Europe. Science. American Association for the Advancement of Science; 2012;336:466–9.

42. Mathieson I, Lazaridis I, Rohland N, Mallick S, Patterson N, Roodenberg SA, *et al.* Genome-wide patterns of selection in 230 ancient Eurasians. Nature. Nature Publishing Group; 2015;528:499–503.

43. Haak W, Paajanen P, Llamas B, Popescu E, Loe L, Clarke R, *et al*. Iron Age and Anglo-Saxon genomes from East England reveal British migration history. Nat. Commun. Nature Publishing Group; 2016;7:1–9.

44. Raghavan M, DeGiorgio M, Albrechtsen A, Moltke I, Skoglund P, Korneliussen TS, *et al.* The genetic prehistory of the New World Arctic. Science. American Association for the Advancement of Science; 2014;345:1255832.

45. Warinner C, Speller C, Collins MJ, Lewis, Jr, Cecil M. Ancient human microbiomes. J. Hum. Evol. Elsevier Ltd; 2015;79:125–36.

46. Warinner C, Rodrigues JFM, Vyas R, Trachsel C, Shved N, Grossmann J, *et al.* Pathogens and host immunity in the ancient human oral cavity. Nat. Genet. Nature Publishing Group; 2014;46:336–44.

47. Bon C, Berthonaud V, Maksud F, Labadie K, Poulain J, Artiguenave F, *et al*. Coprolites as a source of information on the genome and diet of the cave hyena. Proceedings of the Royal Society of London B: Biological Sciences. The Royal Society; 2012;rspb20120358.

48. Tito RY, Knights D, Metcalf J, Obregon-Tito AJ, Cleeland L, Najar F, *et al*. Insights from Characterizing Extinct Human Gut Microbiomes. PLoS One. Public Library of Science; 2012;7:e51146.

49. Fordyce SL, Ávila-Arcos MC, Rasmussen M, Cappellini E, Romero-Navarro JA, Wales N, *et al*. Deep Sequencing of RNA from Ancient Maize Kernels. PLoS One. Public Library of Science; 2013;8:e50961.

50. Keller A, Kreis S, Leidinger P, Maixner F, Ludwig N, Backes C, et al. miRNAs in ancient tissue specimens of the Tyrolean Iceman. Mol. Biol. Evol. [Internet]. 2016; Available from: http://dx.doi.org/10.1093/molbev/msw291

51. Pedersen JS, Valen E, Velazquez AMV, Parker BJ, Rasmussen M, Lindgreen S, *et al.* Genome-wide nucleosome map and cytosine methylation levels of an ancient human genome. Genome Res. 2014;24:454–66.

52. Briggs AW, Stenzel U, Meyer M, Krause J, Kircher M, Paabo S. Removal of deaminated cytosines and detection of in vivo methylation in ancient DNA. Nucleic Acids Res. 2009;1–12.

53. Llamas B, Holland ML, Chen K, Cropley JE, Cooper A, Suter CM. High-resolution analysis of cytosine methylation in ancient DNA. PLoS One. Public Library of Science; 2012;7:e30226.

54. Smith O, Clapham AJ, Rose P, Liu Y, Wang J, Allaby RG. Genomic methylation patterns in archaeological barley show de-methylation as a time-dependent diagenetic process. Sci. Rep. Nature Publishing Group; 2014;4:5559.

55. Gokhman D, Lavi E, Prüfer K, Fraga MF, Riancho JA, Kelso J, *et al*. Reconstructing the DNA methylation maps of the Neandertal and the Denisovan. Science. American Association for the Advancement of Science; 2014;344:523–7.

56. Der Sarkissian C, Allentoft ME, Ávila-Arcos MC, Barnett R, Campos PF, Cappellini E, *et al*. Ancient genomics. Philos. Trans. R. Soc. Lond. B Biol. Sci. 2015;370:20130387.

57. Orlando L, Gilbert MTP, Willerslev E. Reconstructing ancient genomes and epigenomes. Nature Publishing Group. Nature Publishing Group; 2015;16:395–408.

58. Check Hayden E. Is the $1,000 genome for real. Nature News. [Internet] Nature Publishing Group; 2014; Available from: http://www.nature.com/news/is-the-1-000-genome-for-real-1.14530

59. Gilbert MTP, Tomsho LP, Rendulic S, Packard M, Drautz DI, Sher A, *et al*. Whole-genome shotgun sequencing of mitochondria from ancient hair shafts. Science. American Association for the Advancement of Science; 2007;317:1927–30.

60. Pinhasi R, Fernandes D, Sirak K, Novak M, Connell S, Alpaslan-Roodenberg S, *et al.* Optimal Ancient DNA Yields from the Inner Ear Part of the Human Petrous Bone. PLoS One. 2015;10:e0129102.

61. Korlević P, Gerber T, Gansauge M-T, Hajdinjak M, Nagel S, Aximu-Petri A, *et al.* Reducing microbial and human contamination in DNA extractions from ancient bones and teeth. Biotechniques. 2015;59:87–93.

62. Wales N, Andersen K, Cappellini E, Ávila-Arcos MC, Gilbert MTP. Optimization of DNA Recovery and Amplification from Non-Carbonized Archaeobotanical Remains. PLoS One. Public Library of Science; 2014;9:e86827.

63. Dabney J, Knapp M, Glocke I, Gansauge M-T, Weihmann A, Nickel B, *et al*. Complete mitochondrial genome sequence of a Middle Pleistocene cave bear reconstructed from ultrashort DNA fragments. Proc. Natl. Acad. Sci. U. S. A. 2013;110:15758–63.

64. Damgaard PB, Margaryan A, Schroeder H, Orlando L, Willerslev E, Allentoft ME. Improving access to endogenous DNA in ancient bones and teeth. Sci. Rep. 2015;5:11184.

65. Gamba C, Hanghøj K, Gaunitz C, Alfarhan AH, Alquraishi SA, Al-Rasheid KAS, *et al.* Comparing the performance of three ancient DNA extraction methods for high-throughput sequencing. Mol. Ecol. Resour. 2016;16:459–69.

66. Maricic T, Whitten M, Pääbo S. Multiplexed DNA sequence capture of mitochondrial genomes using PCR products. PLoS One. Public Library of Science; 2010;5:e14004.

67. Briggs AW, Good JM, Green RE, Krause J, Maricic T, Stenzel U, *et al*. Primer extension capture: targeted sequence retrieval from heavily degraded DNA sources. J. Vis. Exp. 2009;1573.

68. Carpenter ML, Buenrostro JD, Valdiosera C, Schroeder H, Allentoft ME, Sikora M, *et al.* Pulling out the 1%: Whole-Genome Capture for the Targeted Enrichment of Ancient DNA Sequencing Libraries. Am. J. Hum. Genet. The American Society of Human Genetics; 2013;93:852–64.

69. Burbano HA, Hodges E, Green RE, Briggs AW, Krause J, Meyer M, *et al*. Targeted Investigation of the Neandertal Genome by Array-Based Sequence Capture. Science. American Association for the Advancement of Science; 2010;328:723–5.

70. Enk JM, Devault AM, Kuch M, Murgha YE, Rouillard J-M, Poinar HN. Ancient whole genome enrichment using baits built from modern DNA. Mol. Biol. Evol. 2014;31:1292–4.

71. Seguin-Orlando A, Gamba C, Der Sarkissian C, Ermini L, Louvel G, Boulygina E, *et al.* Pros and cons of methylation-based enrichment methods for ancient DNA. Sci. Rep. Nature Publishing Group; 2015;5:11826.

72. Gansauge M-T, Meyer M. Selective enrichment of damaged DNA molecules for ancient genome sequencing. Genome Res. 2014;24:1543–9.

73. Drmanac R, Sparks AB, Callow MJ, Halpern AL, Burns NL, Kermani BG, *et al*. Human genome sequencing using unchained base reads on self-assembling DNA nanoarrays. Science. American Association for the Advancement of Science; 2010;327:78–81.

74. Fehlmann T, Reinheimer S, Geng C, Su X, Drmanac S, Alexeev A, *et al*. cPAS-based sequencing on the BGISEQ-500 to explore small non-coding RNAs. Clin. Epigenetics. 2016;8:123.

75. Bennike O, Meldgaard M, Heinemeier J, Rud N. Radiocarbon AMS dating of Holocene wolf (*Canis lupus*) remains from Greenland. Holocene. Sage Publications; 1994;4:84–8.

76. Hebsgaard MB, Gilbert MTP, Arneborg J, Heyn P, Allentoft ME, Bunce M, *et al*. "The Farm Beneath the Sand" – an archaeological case study on ancient "dirt" DNA. Antiquity. Cambridge University Press; 2009;83:430–44.

77. Møhl J. Dog Remains from a Paleoeskimo Settlement in West Greenland. Arctic Anthropol. University of Wisconsin Press; 1986;23:81–9.

78. Meldgaard J. Qajâ, en kokkenmodding i dybfrost: Feltrapport fra arbejdsmarken i Gronland. Nationalmuseets Arbejdsmark. 1983. p. 83–96.

79. Grønnow B. Qeqertasussuk-the archaeology of a frozen Saqqaq site in Disko Bugt, West Greenland. Threads Af Arctic Prehistory: Papers in Honour of William E. Taylor Jr. Canadian Museum of Civilization; 1994.

80. Daley T, Smith AD. Predicting the molecular complexity of sequencing libraries. Nat. Methods. Nature Publishing Group; 2013;10:325–7.

81. Ersmark E, Klütsch C, Chan Y, Dalén L, Sinding-Larsen M, Gilbert T, *et al*. From the past to the present: Wolf phylogeography and demographic history based on the mitochondrial control region. 2016 [cited 2017 Jan 13]; Available from: http://www.diva-portal.org/smash/record.jsf?pid=diva2:911453

82. Andrews S, Others. FastQC: a quality control tool for high throughput sequence data. 2010.

83. Schubert M, Lindgreen S, Orlando L. AdapterRemoval v2: rapid adapter trimming, identification, and read merging. BMC Res. Notes. 2016;9:88.

84. McKenna A, Hanna M, Banks E, Sivachenko A, Cibulskis K, Kernytsky A, *et al*. The Genome Analysis Toolkit: a MapReduce framework for analyzing next-generation DNA sequencing data. Genome Res. 2010;20:1297–303.

85. DePristo MA, Banks E, Poplin R, Garimella KV, Maguire JR, Hartl C, *et al*. A framework for variation discovery and genotyping using next-generation DNA sequencing data. Nat. Genet. Nature Publishing Group; 2011;43:491–8.

86. Picard Tools - By Broad Institute [Internet]. [cited 2017 Feb 25]. Available from: https://broadinstitute.github.io/picard/

87. Arnason U, Gullberg A, Janke A, Kullberg M. Mitogenomic analyses of caniform relationships. Mol. Phylogenet. Evol. 2007;45:863–74.

88. Li H. A statistical framework for SNP calling, mutation discovery, association mapping and population genetical parameter estimation from sequencing data. Bioinformatics. 2011;27:2987–93.

89. Li H. Improving SNP discovery by base alignment quality. Bioinformatics. 2011;27:1157–8.

90. lh. GitHub - lh3/seqtk: Toolkit for processing sequences in FASTA/Q formats [Internet]. [cited 2017 Feb 25]. Available from: https://github.com/lh3/seqtk

91. Marçais G, Kingsford C. A fast, lock-free approach for efficient parallel counting of occurrences of k-mers. Bioinformatics. 2011;27:764–70.

92. Benjamini Y, Speed TP. Summarizing and correcting the GC content bias in high-throughput sequencing. Nucleic Acids Res. 2012;40:e72.

93. Alkan C, Kidd JM, Marques-Bonet T, Aksay G, Antonacci F, Hormozdiari F, *et al.* Personalized copy number and segmental duplication maps using next-generation sequencing. Nat. Genet. Nature Publishing Group; 2009;41:1061–7.

94. Smit AFA, Hubley R, Green P. RepeatMasker Open-3.0. 1996.

95. Benson G. Tandem repeats finder: a program to analyze DNA sequences. Nucleic Acids Res. 1999;27:573–80.

96. Marco-Sola S, Sammeth M, Guigó R, Ribeca P. The GEM mapper: fast, accurate and versatile alignment by filtration. Nat. Methods. Nature Publishing Group; 2012;9:1185–8.

 Click here to download Figure Fig1.pdf

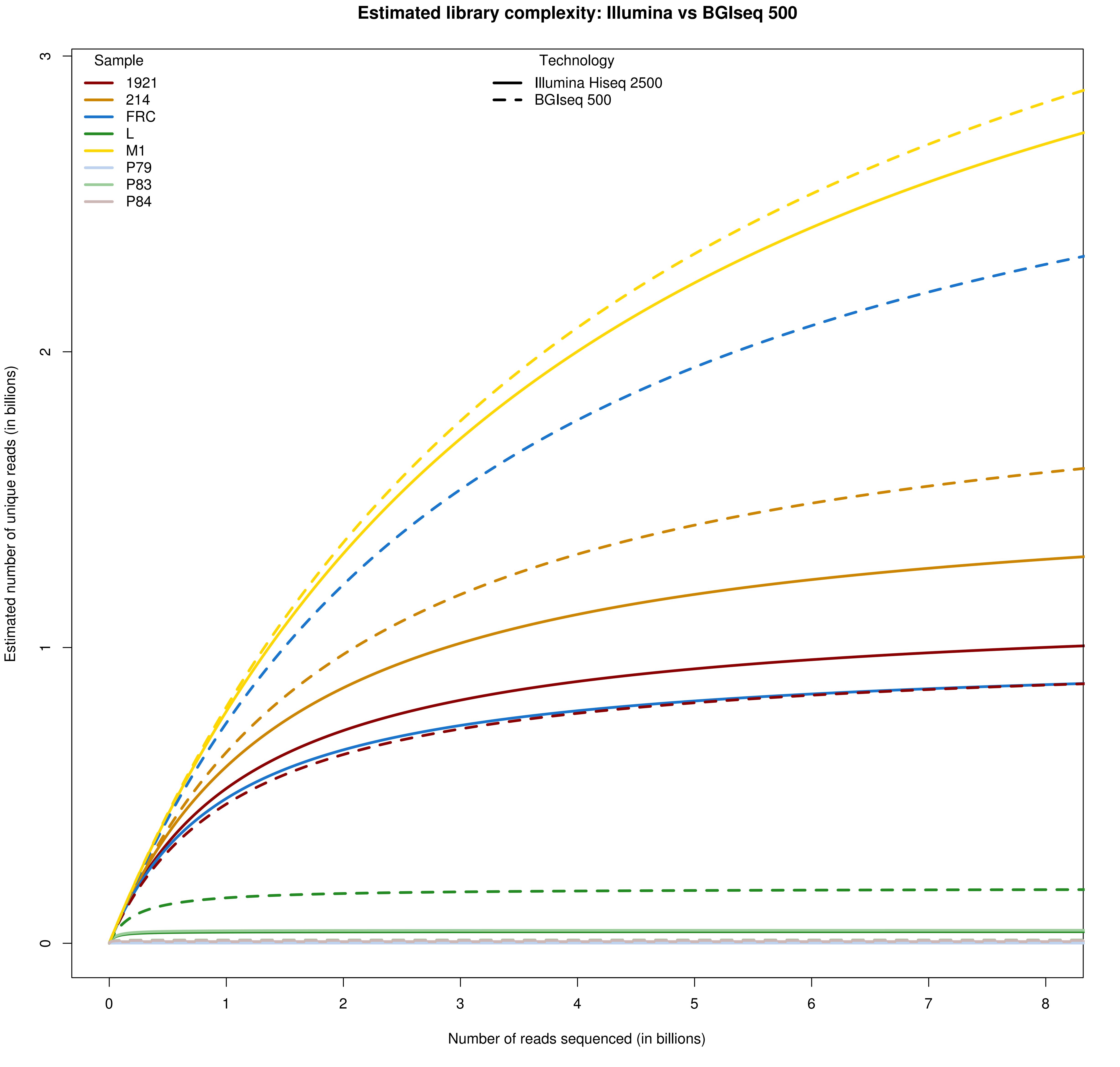

Figure 2

Click here to download Figure Fig2.pdf

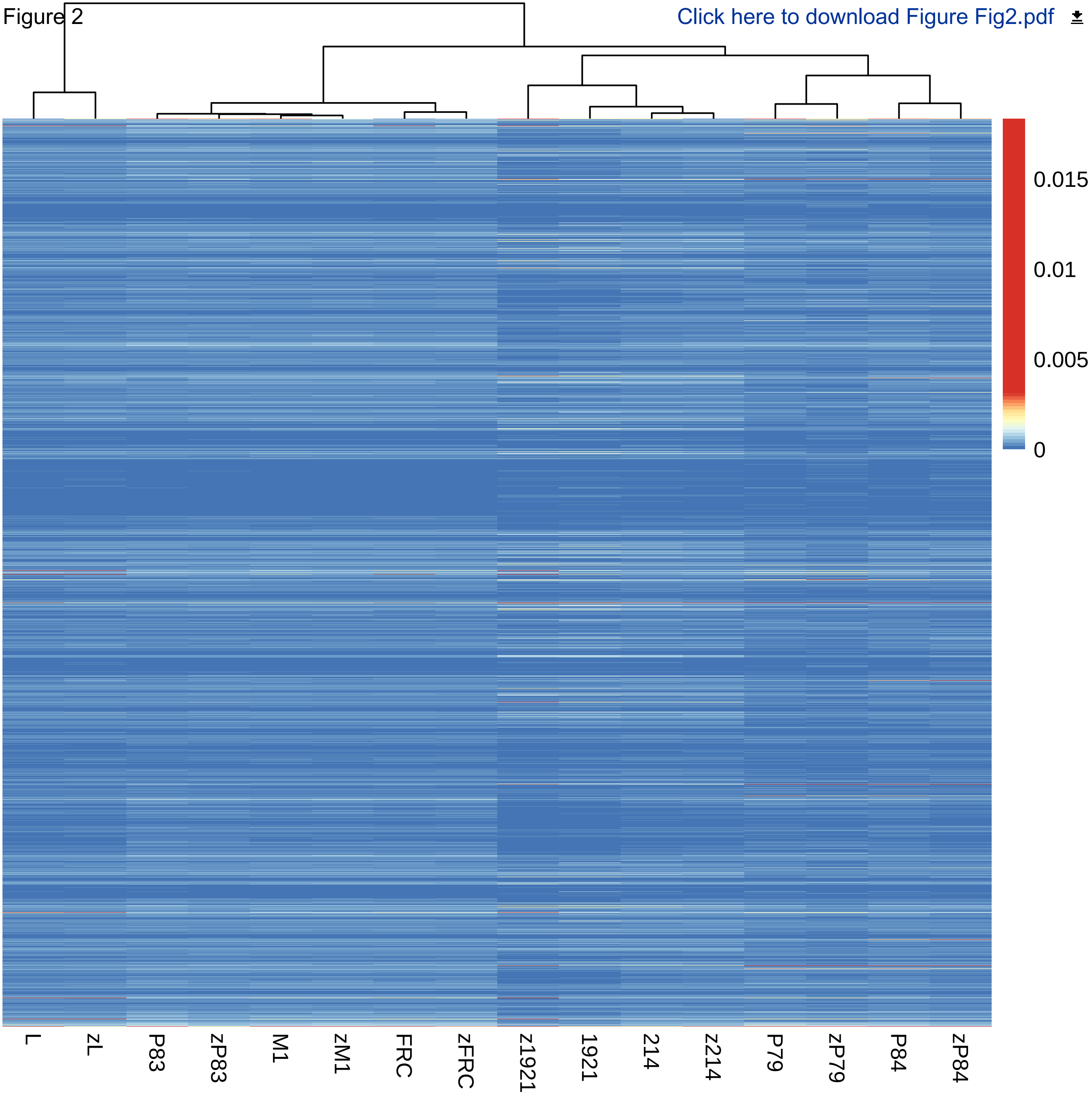

Figure 3

Click here to download Figure Figure_3.png

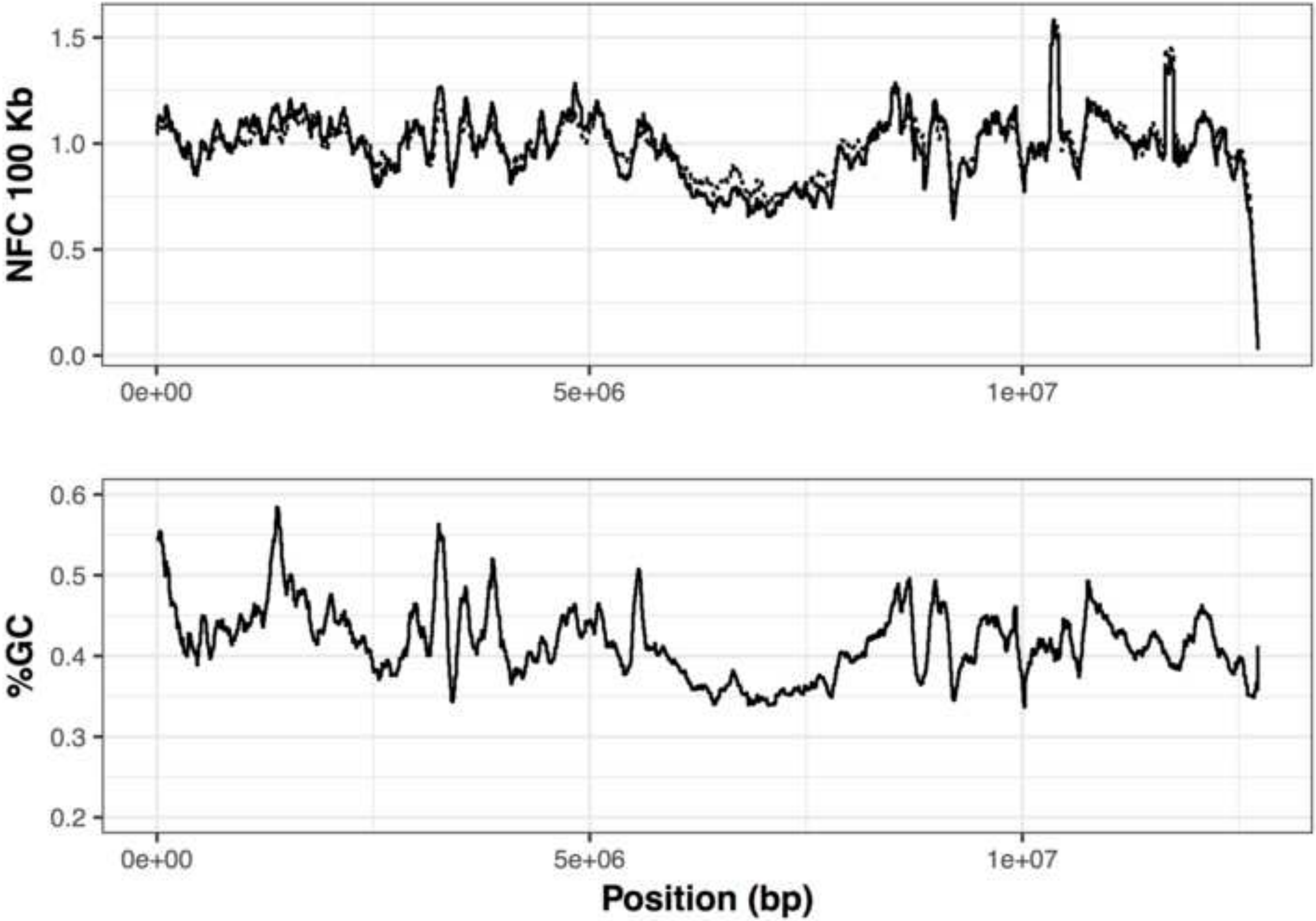

 Click here to download Figure Figure_4.png

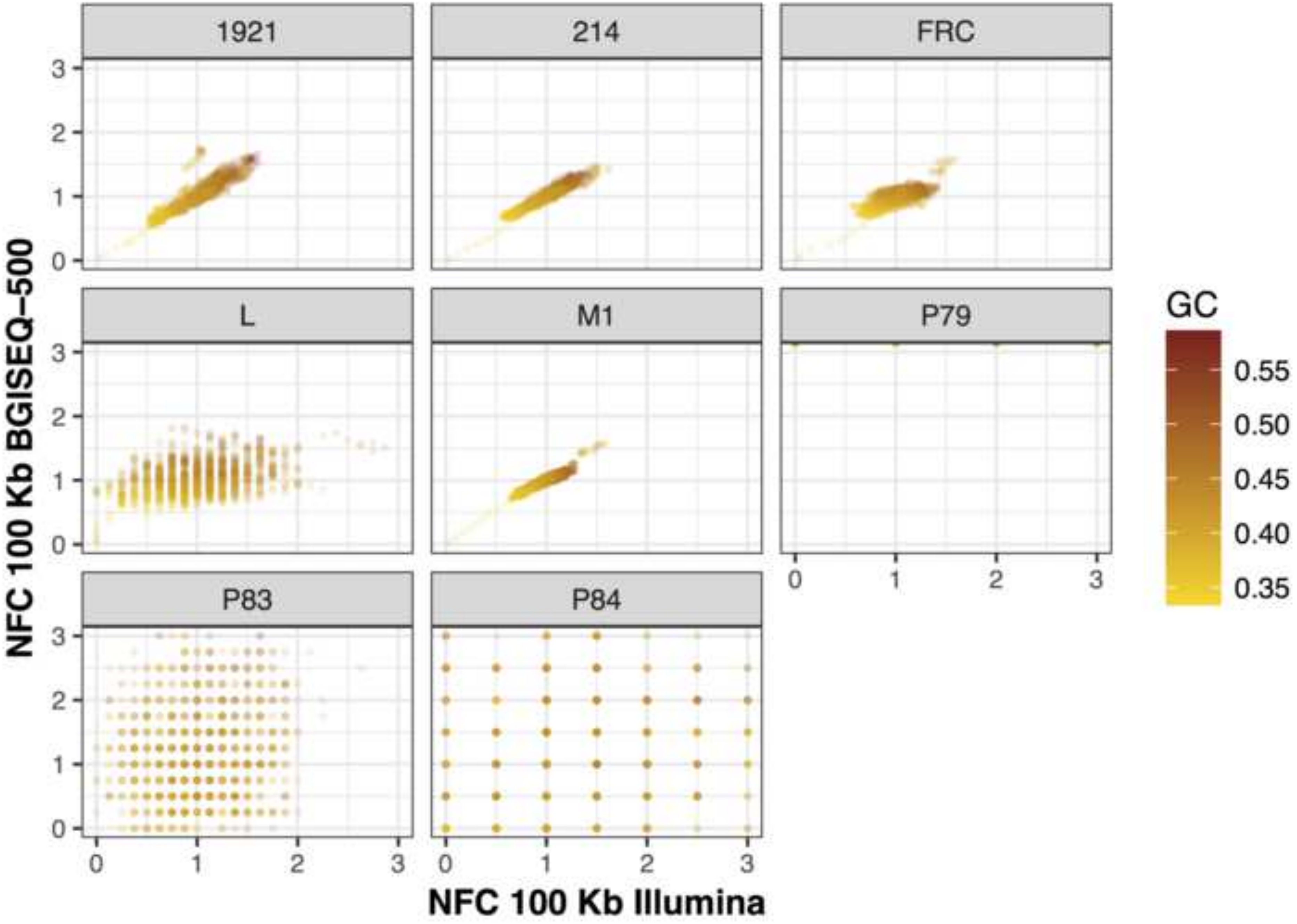

Supplementary Table S1

Click here to access/download
Supplementary Material
Mak_TableS1.xlsx

Supplementary File F1

Click here to access/download
Supplementary Material
Mak_Supplemental File_F1.docx
