# Supplementary material for: Comparative performance of the BGISEQ-500 vs Illumina HiSeq2500 sequencing platforms for palaeogenomic sequencing
Source: Gigascience. 2017 Jun 26;6(8):1–13. doi: 10.1093/gigascience/gix049 (PMC5570000; doi:10.1093/gigascience/gix049)
Supplement: Mak_Supplemental_File_F1.docx [file gix049_mak_supplemental_file_f1.docx]

**Comparative performance of the BGISEQ-500 vs Illumina HiSeq2500 sequencing platforms for palaeogenomic sequencing**

Sarah S.T. Mak *et al.*

**Supplemental File F1 - Improvements to original BEST library building protocol**

To improve the blunt-end single-tube library building as described in Carøe *et al.* (Carøe *et al*., in review), we tested addition of Poly-Ethylene Glycol 4000 (PEG-4000), Bovine Serum Albumin (BSA) and sodium chloride (NaCl) to the end-repair reaction. A so-called ‘Reaction Enhancer’ was prepared containing 25% PEG-4000, 2 μg/μL BSA and 400 mM NaCl to improve the activity of the enzymes in the end repair and downstream reactions. These additives have previously been shown to enhance both activity of T4 Polynucleotide Kinase (T4 PNK) [1,2] and T4 DNA polymerase [3]. Both enzymes are used in the end-repair step of the library building. PEG and salt have also been shown to have a positive effect on activity of the T4 DNA Ligase used in the ligation step [4]. We further tested the effect on the production of library molecules with different amounts of T4 DNA polymerase and T4 PNK. The effect was evaluated using qPCR with C_t_ values indicating of the production of library molecules. We used highly fragmented human genomic DNA as input (average fragment size 100 bp). 70 ng (~1 pmol) was used per reaction and each sample/condition was made in duplicate. Supplemental Table S6 gives an overview of the different conditions, amount of enzyme and addition of Reaction Enhancer.

For the test described above the following protocol was used. The following components were mixed in a 0.5 mL Eppendorf LoBind tube: 70 ng DNA sample (~1 pmol) eluted in 10 mM Tris-HCl, 0.01-0.4 μL T4 DNA polymerase (NEB, 3 U/μL), 0.1-1 μL T4 PNK (NEB, 10 U/μL), 1.6 μL 10x T4 DNA Ligase Buffer (NEB) and 0.3 μL dNTP (Thermo Fischer Scientific, 25 mM) and 10 mM Tris-HCl buffer to reach a final volume of 16 μL. For the reactions that included Reaction Enhancer, one μL of this was used and less Tris-HCl buffer to ensure the same final reaction volume for all samples. The reactions were incubated for 30 minutes at 20 °C followed by 30 minutes at 65 °C. One μL of BEDC3 adapter (10 μM stock) was added and mixed by flicking the tube. See Carøe *et al.* (Carøe *et al*., in review) for details on the BEDC3 adapter for the Illumina platform. A mastermix of 2.5 μL PEG 4000 (Sigma Aldrich, 50%), 0.4 μL T4 DNA Ligase Buffer (10x) and 0.1 μL T4 DNA ligase (NEB, 400 U/μL) was added. The reaction was mixed by flicking the tube and the reaction incubated for 30 minutes at 20 °C followed by 10 minutes at 65 °C. The subsequent fill-in step was performed by adding a mastermix consisting of 0.3 μL dNTP (25 mM stock), 3 μL Isothermal Amplification Buffer 10x (NEB), 6.2 μL molecular biology grade water and 0.5 μL Bst 2.0 Warmstart Polymerase (NEB), directly to the previous reaction. The reaction was incubated at 65 °C for 20 minutes in a prewarmed thermocycler, followed by 20 minutes at 80 °C before cooling to 4 °C. After library build the libraries were purified using Qiagen MinElute columns according to manufacturers recommendations. Quantitative real-time PCR (qPCR) was performed using 1 μL template of a 1:10 dilution of the libraries and using the Roche Lightcycler 480 qPCR mastermix (Roche), 0.2 μM forward and reverse primer (IS7 and IS8 from Meyer & Kircher [5]). qPCR was performed on an Agilent Technologies Mx3005 instrument with the following cycling conditions: 95 °C for 10 minutes, followed by 40 cycles of 95 °C for 30 sec, 60 °C for 30 sec and 72 °C for 1 minute.

**Supplemental Table S6 - Overview of test parameters**

| **Library/sample name** | **T4 PNK (μL)** | **T4 DNA polymerase (μL)** | **Reaction enhancer** |
| --- | --- | --- | --- |
| Control | 0.1 | 0.01 | No |
| High Enzyme | 1 | 0.4 | No |
| Reaction Enhancer | 0.1 | 0.01 | Yes (1 μL) |
| Reaction Enhancer + High Enzyme | 1 | 0.4 | Yes (1 μL) |

The results from qPCR seen in Figure S1 (A) showed that addition of higher enzyme amounts did not seem to affect the production of library molecules in these specific circumstances. It cannot be ruled out that it would have an effect in other circumstances such as higher DNA input amounts. Contrary, adding 1 μL Reaction Enhancer lowered the C_t_ by ~1 cycle. Given the double exponential effect in PCR amplification this roughly translates into 100% more library molecules produced relative to the control. This result was further confirmed when combining the high enzyme amount and the Reaction Enhancer (Figure S1 [A]). Figure S1 (B) shows that the production of adapter dimers dropped when using the Reaction Enhancer. While we do not find an obvious explanation for this observation it should be noted that we in general have observed levels of adapter dimers to vary greatly even within duplicates (data not shown).

**Figure S1. Effect of changing Enzyme amount in the end-repair and adding Reaction Enhancer in a qPCR setup**


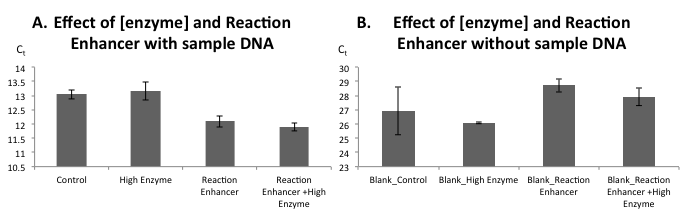


Lower values indicate more efficiency in library build. The specific values for enzyme can be seen in Figure S1. **A)** Effect on 70 ng sample (~1 pmol), **B)** Effect without sample DNA but with adapter. This can be seen as an expression for production of adapter dimers. All samples were done in duplicates. Error bars indicate STD Dev.

**Supplemental References**

1. Lillehaug, J.R., Kleppe, R.K. and Kleppe, K. Phosphorylation of double-stranded DNAs by T4 polynucleotide kinase. Biochemistry. 1976;15(9):1858-65.

2. Harrison, B. & Zimmerman, SB. Stabilization of T4 Polynucleotide Kinase by macromolecular crowding. Anal. Biochem. 1986;158:307.

3. Zimmerman, SB. & Harrison, B. Macromolecular crowding increases binding of DNA polymerase to DNA: An adaptive effect. Proc. Natl. Acad. Sci. USA; 1987;84:1871-1875.

4. Raae, A.J., Kleppe, R.K., & Kleppe, K.. Kinetics and effect of salts and polyamines on T4 Polynucleotide Ligase. Eur. J. Biochem. 1975;60:437-443.

5. Meyer M, Kircher M. Illumina sequencing library preparation for highly multiplexed target capture and sequencing. Cold Spring Harb. Protoc. 2010;2010:db.prot5448.
