# Supplementary material for: Comparative performance of the BGISEQ-500 vs Illumina HiSeq2500 sequencing platforms for palaeogenomic sequencing
Source: Gigascience. 2017 Jun 26;6(8):1–13. doi: 10.1093/gigascience/gix049 (PMC5570000; doi:10.1093/gigascience/gix049)
Supplement: Mak_Tables_S2-S6.pdf [file gix049_mak_tables_s2-s6.pdf]

Comparative performance of the BGISEQ-500 vs Illumina HiSeq2500 sequencing platforms for palaeogenomic sequencing

Sarah S.T. Mak *et al*

Additional Files

Supplemental File F1 – Improvements to original BEST library building protocol

(see additional file)

Supplemental Table S1 - Full sequence data information

(see additional file)

Supplemental Table S2-S6

Supplemental Table S2 - Sequence library identifiers

DNA extract	Illumina Library ID	BGISEQ-500 Library ID (standard)	BGISEQ-500 Library ID (extra purification)
214	214	z_214	z_214p
1921	1921	z_1921	z_1921p
P79	P79	z_P79	z_79p
P83	P83	z_P83	z_P83p
P84	P84	z_P84	z_P84p
FRC	FRC	z_FRC	z_FRCp
L	L	z_L	z_Lp
M1	M1	z_M1	z_M1p

Supplemental Table S3 - The number of index PCR cycles used in each sample

Sample	Platform	Index PCR cycles
1921	Illumina	19
	BGISEQ-500	10
214	Illumina	13
	BGISEQ-500	8
FRC	Illumina	13
	BGISEQ-500	8
L	Illumina	13
	BGISEQ-500	8
M1	Illumina	13
	BGISEQ-500	8
P79	Illumina	19
	BGISEQ-500	10
P83	Illumina	21
	BGISEQ-500	15
P84	Illumina	19
	BGISEQ-500	10

Supplemental Table S4 - The sequences of BGISEQ-500 adapters and index primers used in this study

Name	Sequence (5' -> 3')	Modification
BGISEQ-500 Adapters		
AD1_Long	TTGTCTTCCTAAGACCGCTTGGCCTCCGACTT	
AD1_Short	AAGTCGGAGGCC	
AD2_Long	TTGTCTTCCTAAGGAACGACATGGCTACGATCCGACTT	
AD2_Short	AAGTCGGATCGT	
*Index Primers**		
IndexprimerBGI_1	TGTGAGCCAAGGAGTTG**ACAGTATTTA**TTGTCTTCCTAAGACCGC	
IndexprimerBGI_2	TGTGAGCCAAGGAGTTG**AATTAATTCC**TTGTCTTCCTAAGACCGC	
IndexprimerBGI_3	TGTGAGCCAAGGAGTTG**CTGAGTGACT**TTGTCTTCCTAAGACCGC	
IndexprimerBGI_4	TGTGAGCCAAGGAGTTG**ATTCCGTCAG**TTGTCTTCCTAAGACCGC	
IndexprimerBGI_5	TGTGAGCCAAGGAGTTG**AACTATCTAA**TTGTCTTCCTAAGACCGC	
IndexprimerBGI_6	TGTGAGCCAAGGAGTTG**GGAAGGACCA**TTGTCTTCCTAAGACCGC	
IndexprimerBGI_7	TGTGAGCCAAGGAGTTG**TTATAGAGAG**TTGTCTTCCTAAGACCGC	
IndexprimerBGI_8	TGTGAGCCAAGGAGTTG**GTACAAAGGG**TTGTCTTCCTAAGACCGC	
Commonprimer BGI forward	GAACGACATGGCTACGA	5’ Phosphate

*Variable 10bp indices indicated in bold.

Supplemental Table S5 - Adapter dimer content of initial, and extra purified BGISEQ-500 libraries

Sample	Standard library	Extra purified
1921	82.38%	88.66%
214	99.51%	99.58%
P79	97.47%	98.69%
P83	71.96%	90.90%
P84	87.74%	98.00%
FRC	99.83%	99.72%
L	99.52%	99.95%
M1	99.91%	99.69%

Supplemental Table S6 - Library pooling for BGISEQ-500 library circularisation reactions

Lane number	Pool (ssCir)	Library (Index)
1	ancient_1	Lp (1), M1 (2), 214 (5), FRC (7), others (17-24)
2	ancient_2	P84p (1), M1p (2), 214p (5), 1921 (6), others (9-16)
3	ancient_3	L (1), P83p (2), P79 (3), libCH2* (5), 1921p (6), FRCp (7), others (9-16)
4	ancient_4	P84 (1), P83 (2), P79p (3), libCH1* (4), 214 (5), FRC (7), others (9-16)

*libCH1 and libCH2 are control blank libraries that did not yield any data post sequencing.
