# Supplementary material for: Comparative performance of the BGISEQ-500 vs Illumina HiSeq2500 sequencing platforms for palaeogenomic sequencing
Source: Gigascience. 2017 Jun 26;6(8):1–13. doi: 10.1093/gigascience/gix049 (PMC5570000; doi:10.1093/gigascience/gix049)
Supplement: Response-to-Reviewer-Comments_Original-Submission.pdf [file gix049_response-to-reviewer-comments_original-submission.pdf]

Author's Response To Reviewer Comments

Dear Dr Zauner
Thanks for sending us the reviewer comments and for allowing us to address them. We detail the changes made and our responses otherwise below. We hope you find them acceptable.

We would also like to highlight one change to the manuscript. In preparing the original text we inadvertently left of the sample providers as authors due to a miscommunication between us and then. We apologise for this error, but have added them (Germonpre, Bocherens, Fedorov). to the resubmission. Given their critical role in provenancing the material, we feel it is fair to them and hope you do not object.

Best wishes
Tom Gilbert and colleagues.

Editor comments:

Reviewer 2 has some remarks regarding reporting of statistical parameters that I feel are quite important. Also, answering the reviewer's question regarding performance on modern samples, in comparison to historic DNA, will add value to your manuscript.

*As we hope you'll see below, we have addressed these issues.

Reviewer 3 suggests you explore the possibility that the rate of sequencing error may be slightly higher in the BGISEQ, and the reviewer presents some ideas how this could be tested.

*We respond to this comment under replies to Rev 3.

On a related note, you may be interested that we have just published another paper on the BGISEQ technology:
https://academic.oup.com/gigascience/article/3098240/A-reference-human-genome-dataset-of-the-BGISEQ-500

*We have now added this information into the abstract and elsewhere.

Reviewer reports:
Reviewer #1: This is a methods paper which investigates the use of an alternative sequencing platform for paleogenomic investigation. The state of the art is well-reviewed and the case for the importance of this is well made. The topic is novel and to my knowledge has not been investigated elsewhere.

On the whole, the work is solid and of interest - an alternative to Illumina platforms, if proved viable as implicated here, could help introduce some much needed competition into this market. My own laboratory's work, for example, could benefit from this. The statistics used are suitably informative and the manuscript is clearly written. However, a couple of further comments.

The variance in mtDNA sequence recovery % between samples is noted and suggested as related to endogenous DNA amounts. I suggest that a major determinant here is the different preservation biases between bone and soft tissue for ancient remains.

*We agree with this, however feel our text must have been unclear. Our analyses related to the platform specific differences, not the inter-sample differences. We have now modified the wording to make this hopefully more clear.

Whereas the case is made that the characteristics of Illumina and BGIseq-500 derived sequence are equivalent. However, can the authors argue that this shall hold for analyses involving whole genome summary statistics? If I am not mistaken, this new technology is built upon the Complete Genomics technology acquired by BGI. There is a high quality data set of multiple human genomes published by Complete Genomics that have been the focus of several investigations. However, I am not aware that these have been co-analysed by the community in combination with equivalent emerging Illumina-derived genomes, despite their obvious interest. I think some discussion on this would help allay fears that BGISeq-500 data will be fully compatible with legacy Illumina genomes if practitioners are motivated to switch because of cost or other factors.

*We agree with this point completely. However unfortunately as our data is not high enough sequence coverage to make accurate statements on this point, we can not address it here. We have however added extra text to the end of the ‘Potential Implications’ to briefly mention this point, and in particular suggesting that this will be a valuable extra area for exploration as more BGIseq data begins to appear. Specifically:

*We do caution however, that due to the small size of the dataset (both sample numbers and sequencing depth), at this point we are not able to offer any comment as to how this overall evidence of consistency may translate into downstream analyses involving whole genome summary statistics. Thus we strongly advocate that those who may be interested in using the BGISEQ platform in population genomic explore this point further.’

Reviewer #2: Sara Siu Tze Mak et al. presented an interesting manuscript on comparison between the established Illumina sequencing platform and novel BGISEQ in application for aDNA analysis. The authors carefully describe experimental and data processing details and test the comparison on a range of samples, from ancient to historic. The only missing component is comparison of accuracy for modern dog/wolf samples and confirmation of various statement by p-values.

These are my comments:

1. Vague statements - such as (line 50 in the abstract) "the data generated was largely comparable between sequencing platforms, with no statistically significant differences". I recommend being more specific, and provide p-values for comparison

*P values are now added to the abstract as requested.

2. Lines 56-57 of the abstract contains a statement that about significant difference detected between levels of sequences mtDNA, and the explanation provided is that there is a very low level of endogenous material. It is a bit puzzling, since nuclear DNA degrades at least twice as fast as mtDNA (Allentoft et al., 2012). Rizzi et al (2012) noted that since mtDNA sequences occur in many hundreds of copies per cell, they "are more easily retrieved from ancient specimens than are nuclear DNA sequences that occur only once per haploid genome." Therefore, I suggest to clarify the statement about the sequences of mtDNA.

*We have clarified this wording as requested, to indicate we believe this relates to the fact that we recovered very few total endogenous DNA reads, and thus fewer still mtDNA reads from 3 samples, thus it is likely due to stochasticity.

3. In the background section (line 67) I suggest that it is not a good idea to refer to NGS as "so-called". NGS is an established term

*Thanks, change now made.

4. Before using abbreviations, such as mtDNA, nuDNA, mitogenome (lines 69-71), make sure to introduce and, if needed, to explain the term

*Thanks, change now made.

5. Lines 72-72 contain a statement "near-complete ancient nuclear genomes (so-called palaeogenomes)" is confusing. Not only nuclear genomes extracted from ancient remains are called palaeogenomes -this term can also apply to mtDNA. And if the genome happens to be complete - is it palaeogenome in this case or not?

*It is impossible to generate complete nuclear genomes from ancient samples (and indeed from many modern samples) due to the large repeat regions that they contain. Thus the only sensible use of the term palaeogenome is when genome scale data is generated. Similar to how the term genome these days refers to relatively complete, but not complete, genomes. We have clarified the wording to read 'However, thanks to NGS techniques, with the right sample and sufficient funds, today practitioners are able to aim for relatively complete ancient nuclear genomes (hereafter referred to as palaeogenomes),'

6. In lines 76/77 please add that Minion and PacBio are not used for aDNA also due to higher error rate, in addition to other reasons.

*We disagree with making this change, thus have not done it. In theory, error rate can be accommodated if samples are sequenced at depth, and indeed we are aware of groups trying to apply both platforms to aDNA (not that we don't agree with Rev 2 that its largely an inefficient exercise trying to apply Minion and PacBio to aDNA).

7. Lines 78-79. Discussion of Roche/454 could be omitted, since both methods have been made obsolete. It was announced in 2013 that "Roche is shuttering its 454 Life Sciences sequencing

operations and laying off about 100 employees...The 454 sequencers will be phased out in mid-2016, and the 454 facility in Branford, Conn., will be closed" (https://www.genomeweb.com/sequencing/roche-shutting-down-454-sequencing-business).

*While we agree they are being shut down, we are aware of groups that are still using them for palaeogenomics, thus we have left this in.

8. Line 81 - specify "acceptable" sequencing error rate

*Done

9. Lines 83-85 has several problems. In the sentence ", has been a considerable focus…" possibly the word "there" is missing in the beginning. In addition, "considerable focus" is probably bad English.

*Thanks for noticing this. Now fixed.

10. In the lines 78-93, the authors are going back and forth between method, repeatedly stating that Illumina is the preferred work horse. It would be sufficient to say that Illumina is an instrument of choice and give references to QC papers.

*We have modified the text slightly, but would like to maintain the methods references as we feel this is an important point.

11. Line 100. "The per base cost of Illumina-based NGS sequencing" sounds bad due to repeated use of "base". If the author accepts my suggestion about restricting the discussion to Illumina, they would not have to specify that it is Illumina-based.

*This has now been modified.

12. Line 103. $1000 per genome was announced several years ago. May be, it worth mentioning newer target for the price tag $100 per genome (https://www.forbes.com/sites/matthewherper/2017/01/09/illumina-promises-to-sequence-human-genome-for-100-but-not-quite-yet/2/#6a7250c66ea4)

*While we appreciate this comment, we are extremely sceptical of company price claims in the media, and furthermore the conventional commercial cost with companies such as Novogene are still >USD 1000 per genome (at 30x coverage). Thus we would like to keep the 1000 USD sum mentioned.

13. Lines 104-105. Please provide range for external content.

*We have not done that, as to be frank it ranges from 100% non endogenous DNA in the worst case, to 0% in the best case. Thus speficying a range seems somewhat unhelpful.

14. Line 117. refer to the comparison between BGISEQ and Illumina

(https://www.genomeweb.com/sequencing/researchers-compare-bgiseq-500-hiseq-microarray-platforms-sequencing-microrna, http://www.nature.com/nrg/journal/v17/n6/fig_tab/nrg.2016.49_T1.html)

*We have added the Nature Reviews Genetics citation, thanks for the suggestion.

15. Prior to analysis of ancient DNA, it would make sence to compare BGISEQ and Illumina using modern dog DNA.

*This has been done on modern DNA, thus we have now highlighted this in the text (discussion with the editor indicated this was an appropriate solution).

16. Line 128 - Need to specify degree of DNA degradation/preservation.

*This information is present in Table 1 and 2 so we have not added it again.

17. Line 174 - please explain why different number of reads was generated for Illumina and BGISEQ.

*This different platforms have very different sequence outputs, and further when pooling samples it is nearly impossible to hit identical levels of sequencing. However it is because of this variation that our analyses are done on both the full data, and normalised data, as discussed in the appropriate other parts of the manuscript. Thus we feel that this point is not worth elaborating on. Should the editor agree then we can add a note.

18. In the lines 192-200 please specify exact values of damage rate, PCR cycles and differences from the reference genome.

*Details of PCR cycles are added in Supplemental Table S3 and the other information is present in Table 2.

19. In the lines 205-208 the authors suggest that differences of regions of genomes sequence could be driving the difference between the BGISEQ and Illumina performance for one of the samples. This hypothesis could be statistically tested.

*While we agree that this could be the case, as stated in the subsequent lines, with our low coverage we do not feel we can test this at the current point (note that we would be restricted to analysing data normalised to the lowest level of sequence data that we have for each or our test pairs). Should the referee believe that we are incorrect and it is testable, we are open to any suggestions that the reviewer might have as to how to test it, given our data. We also highlight that reconsideration of the text lead us to conclude that the wording could be optimised, thus this section now reads: “Alternatively, we hypothesise that an alternative explanation for the observed differences in δS and θ might relate to the relatively low genome coverage that we have for each sample. As such, each sample was sequenced over different parts of the genome, which in turn may lead to small biases in the error profiles. Ultimately however, we feel that full resolution of the differences will require the generation of extensive extra data, and thus more

will be learnt in future studies that use the BGISEQ-500."

20. Line 241-244. The dilemma can be resolved by repeating the correlation calculation imposing a strict coverage cut-off.

*We thank the reviewer for pointing this out. We recalculated correlations coefficients of fragment counts in 100 Kb windows after randomly down-sampling the higher coverage platform to the same number of high-quality mapped bases of the lower coverage platform. The results of this analysis are now reflected in Table 4, as well as Figures 3 & 4. We have furthermore decided to drop the numbers of correlation coefficients for windows on scaffold_0 only. We originally calculated those values to avoid any potential biases poorly assembled regions of the genome might introduce, as the genome wide correlations of fragment count were below our expectation. Given that the genome wide correlation coefficients now match our expectations of comparable performance for the two platforms, we feel it is unnecessary to further include them.

21. Lines 431-440. It would be interesting to see how selection of aligner and variant caller affects the concordance between BGISEQ and Illumina. However, it may be beyond the scope of the current paper.

*We agree with this, but also feel it is beyond the scope of our paper, however the point is important, thus we now mention this in the 'Potential Implications' section. Specifically we have added text to say: "Furthermore, as additional datasets are generated, we look forward to the results of analyses that might wish to compare the relative performance of different sequence alignment and variant calling software on such data."

Reviewer #3: This study investigates the use of the new BGISEQ-500 platform for palaeogenomics. To do so, the authors compare data obtained from both BGISEQ and Illumina Hi-Seq. The current version of manuscript is well written, concise and its conclusions are both reasonable and well founded. This study adds an interesting perspective for future palaeogenomics experiments.

I have only a few comments that I hope will improve the manuscript.

Major

While I can see that the difference in θ is not so worrying, I am not so convinced by the authors' interpretation. Instead I am wondering whether the difference could due to slightly higher rate of sequencing error in the BGISEQ-500. I think this could be tested using duplicates. Comparing the rate of mismatch between duplicates (defined as reads starting and ending at same position) for each platform could be informative about sequencing error?

*We feel this comment may arise due to a miscommunication. We state in the "Potential Implications" section that we also believe that the difference in the estimate of theta is

potentially driven by higher sequencing error rates on the BGI platform. Further, the methods suggested by the reviewer about using the duplicate reads within each sample to estimate the sequencing error is very underpowered here due to both the low number of reads per samples (low coverages) and low number of PCR duplicates for samples with higher coverages.

Another approach would be to compare the rate of mismatch, to the reference genome, of same molecules sequenced by the Hi-Seq and BGISEQ-500 platforms (cross platform duplicates). This second approach might be hard given the low coverage but the authors might have enough reads to do this on some samples.

*The reviewer is correct in assessing that due to coverage issues, the number of “duplicate” reads across platforms are very low, so it is impossible to compute the cross-platform discordance. Additionally, this approach does not indicate which of the two platforms had the error, only that one of them did, since the bases differ from each other, making this estimate a bit difficult to interpret.

Minor

Use conventional cal BP for calibrated dates.

*We have added this to the appropriate dates. (Please note not all dates are 14C dates)

Table 2: Coloring rows for the same samples or using bold lines to separate samples would help the readability of this table.

*Done

The "relative abundance versus GC content" section in the method could use some editing for clarity.

*Thanks for the comment, now done.
