# Supplementary material for: Comparative performance of the BGISEQ-500 vs Illumina HiSeq2500 sequencing platforms for palaeogenomic sequencing
Source: Gigascience. 2017 Jun 26;6(8):1–13. doi: 10.1093/gigascience/gix049 (PMC5570000; doi:10.1093/gigascience/gix049)
Supplement: Reviewer-1-Report-(Original-Submission).pdf [file gix049_reviewer-1-report-(original-submission).pdf]

Reviewer Report

Title: Comparative performance of the BGISEQ-500 versus Illumina HiSeq2500 sequencing platforms for palaeogenomic sequencing

Version: Original Submission **Date:** 13 Mar 2017

Reviewer name: Daniel Bradley

Reviewer Comments to Author:

This is a methods paper which investigates the use of an alternative sequencing platform for paleogenomic investigation. The state of the art is well-reviewed and the case for the importance of this is well made. The topic is novel and to my knowledge has not been investigated elsewhere.

On the whole, the work is solid and of interest - an alternative to Illumina platforms, if proved viable as implicated here, could help introduce some much needed competition into this market. My own laboratory's work, for example, could benefit from this. The statistics used are suitably informative and the manuscript is clearly written. However, a couple of further comments.

The variance in mtDNA sequence recovery % between samples is noted and suggested as related to endogenous DNA amounts. I suggest that a major determinant here is the different preservation biases between bone and soft tissue for ancient remains.

Whereas the case is made that the characteristics of Illumina and BGIseq-500 derived sequence are equivalent. However, can the authors argue that this shall hold for analyses involving whole genome summary statistics? If I am not mistaken, this new technology is built upon the Complete Genomics technology acquired by BGI. There is a high quality data set of multiple human genomes published by Complete Genomics that have been the focus of several investigations. However, I am not aware that these have been co-analysed by the community in combination with equivalent emerging Illumina-derived genomes, despite their obvious interest. I think some discussion on this would help allay fears that BGISeq-500 data will be fully compatible with legacy Illumina genomes if practitioners are motivated to switch because of cost or other factors.

Methods

Are the methods appropriate to the aims of the study, are they well described, and are necessary controls included? Yes

Conclusions

Are the conclusions adequately supported by the data shown? Yes

Reporting Standards

Does the manuscript adhere to the journal's guidelines on minimum standards of reporting? Yes

Statistics

Are you able to assess all statistics in the manuscript, including the appropriateness of statistical tests used? Yes, and I have assessed the statistics in my report.

Quality of Written English

Please indicate the quality of language in the manuscript: Acceptable

Declaration of Competing Interests

Please complete a declaration of competing interests, considering the following questions:

- Have you in the past five years received reimbursements, fees, funding, or salary from an organisation that may in any way gain or lose financially from the publication of this manuscript, either now or in the future?
- Do you hold any stocks or shares in an organisation that may in any way gain or lose financially from the publication of this manuscript, either now or in the future?
- Do you hold or are you currently applying for any patents relating to the content of the manuscript?
- Have you received reimbursements, fees, funding, or salary from an organization that holds or has applied for patents relating to the content of the manuscript?
- Do you have any other financial competing interests?
- Do you have any non-financial competing interests in relation to this paper?

If you can answer no to all of the above, write 'I declare that I have no competing interests' below. If your reply is yes to any, please give details below.

I declare that I have no competing interests

I agree to the open peer review policy of the journal. I understand that my name will be included on my report to the authors and, if the manuscript is accepted for publication, my named report including any attachments I upload will be posted on the website along with the authors' responses. I agree for my report to be made available under an Open Access Creative Commons CC-BY license (http://creativecommons.org/licenses/by/4.0/). I understand that any comments which I do not wish to be included in my named report can be included as confidential comments to the editors, which will not be published.

I agree to the open peer review policy of the journal

To further support our reviewers, we have joined with Publons, where you can gain additional credit to further highlight your hard work (see: https://publons.com/journal/530/gigascience). On publication of this paper, your review will be automatically added to Publons, you can then choose whether or not to claim your Publons credit. I understand this statement.

Yes
