# Supplementary material for: Comparative performance of the BGISEQ-500 vs Illumina HiSeq2500 sequencing platforms for palaeogenomic sequencing
Source: Gigascience. 2017 Jun 26;6(8):1–13. doi: 10.1093/gigascience/gix049 (PMC5570000; doi:10.1093/gigascience/gix049)
Supplement: Reviewer-2-Report-(Original-Submission).pdf [file gix049_reviewer-2-report-(original-submission).pdf]

Reviewer Report

Title: Comparative performance of the BGISEQ-500 versus Illumina HiSeq2500 sequencing platforms for palaeogenomic sequencing

Version: Original Submission **Date:** 22 Mar 2017

Reviewer name: Tatiana Tatarinova

Reviewer Comments to Author:

Sara Siu Tze Mak et al. presented an interesting manuscript on comparison between the established Illumina sequencing platform and novel BGISEQ in application for aDNA analysis. The authors carefully describe experimental and data processing details and test the comparison on a range of samples, from ancient to historic. The only missing component is comparison of accuracy for modern dog/wolf samples and confirmation of various statement by p-values.

These are my comments:

1. Vague statements - such as (line 50 in the abstract) "the data generated was largely comparable between sequencing platforms, with no statistically significant differences". I recommend being more specific, and provide p-values for comparison

2. Lines 56-57 of the abstract contains a statement that about significant difference detected between levels of sequences mtDNA, and the explanation provided is that there is a very low level of endogenous material. It is a bit puzzling, since nuclear DNA degrades at least twice as fast as mtDNA (Allentoft et al., 2012). Rizzi et al (2012) noted that since mtDNA sequences occur in many hundreds of copies per cell, they "are more easily retrieved from ancient specimens than are nuclear DNA sequences that occur only once per haploid genome." Therefore, I suggest to clarify the statement about the sequences of mtDNA.

3. In the background section (line 67) I suggest that it is not a good idea to refer to NGS as "so-called". NGS is an established term

4. Before using abbreviations, such as mtDNA, nuDNA, mitogenome (lines 69-71), make sure to introduce and, if needed, to explain the term

5. Lines 72-72 contain a statement "near-complete ancient nuclear genomes (so-called palaeogenomes)" is confusing. Not only nuclear genomes extracted from ancient remains are called palaeogenomes -this term can also apply to mtDNA. And if the genome happens to be complete - is it palaeogenome in this case or not?

6. In lines 76/77 please add that Minion and PacBio are not used for aDNA also due to higher error rate, in addition to other reasons.

7. Lines 78-79. Discussion of Roche/454 could be omitted, since both methods have been made obsolete. It was announced in 2013 that "Roche is shuttering its 454 Life Sciences sequencing operations and laying off about 100 employees...The 454 sequencers will be phased out in mid-2016, and the 454 facility in Branford, Conn., will be closed" (https://www.genomeweb.com/sequencing/roche-shutting-down-454-sequencing-business).

8. Line 81 - specify "acceptable" sequencing error rate

9. Lines 83-85 has several problems. In the sentence ", has been a considerable focus…" possibly the word "there" is missing in the beginning. In addition, "considerable focus" is probably bad English.

10. In the lines 78-93, the authors are going back and forth between method, repeatedly stating that Illumina is the preferred work horse. It would be sufficient to say that Illumina is an instrument of choice and give references to QC papers.

11. Line 100. "The per base cost of Illumina-based NGS sequencing" sounds bad due to repeated use of "base". If the author accepts my suggestion about restricting the discussion to Illumina, they would not have to specify that it is Illumina-based.

12. Line 103. $1000 per genome was announced several years ago. May be, it worth mentioning newer target for the price tag $100 per genome (https://www.forbes.com/sites/matthewherper/2017/01/09/illumina-promises-to-sequence-human-genome-for-100-but-not-quite-yet/2/#6a7250c66ea4)

13. Lines 104-105. Please provide range for external content.

14. Line 117. refer to the comparison between BGISEQ and Illumina (https://www.genomeweb.com/sequencing/researchers-compare-bgiseq-500-hiseq-microarray-platforms-sequencing-microrna, http://www.nature.com/nrg/journal/v17/n6/fig_tab/nrg.2016.49_T1.html)

15. Prior to analysis of ancient DNA, it would make sence to compare BGISEQ and Illumina using modern dog DNA.

16. Line 128 - Need to specify degree of DNA degradation/preservation.

17. Line 174 - please explain why different number of reads was generated for Illumina and BGISEQ.

18. In the lines 192-200 please specify exact values of damage rate, PCR cycles and differences from the

reference genome.

19. In the lines 205-208 the authors suggest that differences of regions of genomes sequence could be driving the difference between the BGISEQ and Illumina performance for one of the samples. This hypothesis could be statistically tested.

20. Line 241-244. The dilemma can be resolved by repeating the correlation calculation imposing a strict coverage cut-off.

21. Lines 431-440. It would be interesting to see how selection of aligner and variant caller affects the concordance between BGISEQ and Illumina. However, it may be beyond the scope of the current paper.

Methods

Are the methods appropriate to the aims of the study, are they well described, and are necessary controls included? Yes

Conclusions

Are the conclusions adequately supported by the data shown? Yes

Reporting Standards

Does the manuscript adhere to the journal's guidelines on [minimum standards of reporting?] Yes

Statistics

Are you able to assess all statistics in the manuscript, including the appropriateness of statistical tests used? Yes, and I have assessed the statistics in my report.

Quality of Written English

Please indicate the quality of language in the manuscript: Acceptable

Declaration of Competing Interests

Please complete a declaration of competing interests, considering the following questions:

- Have you in the past five years received reimbursements, fees, funding, or salary from an organisation that may in any way gain or lose financially from the publication of this manuscript, either now or in the future?
- Do you hold any stocks or shares in an organisation that may in any way gain or lose financially from the publication of this manuscript, either now or in the future?

- Do you hold or are you currently applying for any patents relating to the content of the manuscript?
- Have you received reimbursements, fees, funding, or salary from an organization that holds or has applied for patents relating to the content of the manuscript?
- Do you have any other financial competing interests?
- Do you have any non-financial competing interests in relation to this paper?

If you can answer no to all of the above, write 'I declare that I have no competing interests' below. If your reply is yes to any, please give details below.

I declare I have no competing interests

I agree to the open peer review policy of the journal. I understand that my name will be included on my report to the authors and, if the manuscript is accepted for publication, my named report including any attachments I upload will be posted on the website along with the authors' responses. I agree for my report to be made available under an Open Access Creative Commons CC-BY license (http://creativecommons.org/licenses/by/4.0/). I understand that any comments which I do not wish to be included in my named report can be included as confidential comments to the editors, which will not be published.

I agree to the open peer review policy of the journal

To further support our reviewers, we have joined with Publons, where you can gain additional credit to further highlight your hard work (see: https://publons.com/journal/530/gigascience). On publication of this paper, your review will be automatically added to Publons, you can then choose whether or not to claim your Publons credit. I understand this statement.

Yes
