# Supplementary material for: Comparative performance of the BGISEQ-500 vs Illumina HiSeq2500 sequencing platforms for palaeogenomic sequencing
Source: Gigascience. 2017 Jun 26;6(8):1–13. doi: 10.1093/gigascience/gix049 (PMC5570000; doi:10.1093/gigascience/gix049)
Supplement: Reviewer-3-Report-(Original-Submission).pdf [file gix049_reviewer-3-report-(original-submission).pdf]

Reviewer Report

Title: Comparative performance of the BGISEQ-500 versus Illumina HiSeq2500 sequencing platforms for palaeogenomic sequencing

Version: Original Submission **Date:** 29 Mar 2017

Reviewer name: Laurent Frantz

Reviewer Comments to Author:

This study investigates the use of the new BGISEQ-500 platform for palaeogenomics. To do so, the authors compare data obtained from both BGISEQ and Illumina Hi-Seq. The current version of manuscript is well written, concise and its conclusions are both reasonable and well founded. This study adds an interesting perspective for future palaeogenomics experiments.

I have only a few comments that I hope will improve the manuscript.

Major

While I can see that the difference in θ is not so worrying, I am not so convinced by the authors' interpretation. Instead I am wondering whether the difference could due to slightly higher rate of sequencing error in the BGISEQ-500. I think this could be tested using duplicates. Comparing the rate of mismatch between duplicates (defined as reads starting and ending at same position) for each platform could be informative about sequencing error? Another approach would be to compare the rate of mismatch, to the reference genome, of same molecules sequenced by the Hi-Seq and BGISEQ-500 platforms (cross platform duplicates). This second approach might be hard given the low coverage but the authors might have enough reads to do this on some samples.

Minor

Use conventional cal BP for calibrated dates.

Table 2: Coloring rows for the same samples or using bold lines to separate samples would help the readability of this table.

The "relative abundance versus GC content" section in the method could use some editing for clarity.

Methods

Are the methods appropriate to the aims of the study, are they well described, and are necessary controls included? Yes

Conclusions

Are the conclusions adequately supported by the data shown? Yes

Reporting Standards

Does the manuscript adhere to the journal's guidelines on [minimum standards of reporting?] Yes

Statistics

Are you able to assess all statistics in the manuscript, including the appropriateness of statistical tests used? Yes, and I have assessed the statistics in my report.

Quality of Written English

Please indicate the quality of language in the manuscript: Acceptable

Declaration of Competing Interests

Please complete a declaration of competing interests, considering the following questions:

- Have you in the past five years received reimbursements, fees, funding, or salary from an organisation that may in any way gain or lose financially from the publication of this manuscript, either now or in the future?
- Do you hold any stocks or shares in an organisation that may in any way gain or lose financially from the publication of this manuscript, either now or in the future?
- Do you hold or are you currently applying for any patents relating to the content of the manuscript?
- Have you received reimbursements, fees, funding, or salary from an organization that holds or has applied for patents relating to the content of the manuscript?
- Do you have any other financial competing interests?
- Do you have any non-financial competing interests in relation to this paper?

If you can answer no to all of the above, write 'I declare that I have no competing interests' below. If your reply is yes to any, please give details below.

I declare that I have no competing interests

I agree to the open peer review policy of the journal. I understand that my name will be included on my report to the authors and, if the manuscript is accepted for publication, my named report including any attachments I upload will be posted on the website along with the authors' responses. I agree for my report to be made available under an Open Access Creative Commons CC-BY license (http://creativecommons.org/licenses/by/4.0/). I understand that any comments which I do not wish to

be included in my named report can be included as confidential comments to the editors, which will not be published.

I agree to the open peer review policy of the journal

To further support our reviewers, we have joined with Publons, where you can gain additional credit to further highlight your hard work (see: https://publons.com/journal/530/gigascience). On publication of this paper, your review will be automatically added to Publons, you can then choose whether or not to claim your Publons credit. I understand this statement.

Yes
